# Rapid and direct discovery of functional tumor specific neoantigens by high resolution mass spectrometry and novel algorithm prediction

**DOI:** 10.1016/j.cellin.2025.100251

**Published:** 2025-05-12

**Authors:** Huajian Tian, Guifei Li, Cookson K.C. Chiu, E. Li, Yuzong Chen, Ting Zhu, Min Hu, Yanjie Wang, Suping Wen, Jiajia Li, Shuangxue Luo, Zhicheng Chen, Huimei Zeng, Nan Zheng, Jinyong Wang, Weijun Shen, Xi Kang

**Affiliations:** a Translation Innovation center, Shenzhen Bay Laboratory, Shenzhen 518132, Guangdong, China; b Multi-omics Mass Spectrometry Core, Biomedical Research Core Facilities, Shenzhen Bay Laboratory, Shenzhen 518132, Guangdong, China; c Genomics core, Biomedical Research Core Facilities, Shenzhen Bay Laboratory, Shenzhen 518132, Guangdong, China; d Institute of Biomedical Health Technology and Engineering, Shenzhen Bay Laboratory, Shenzhen 518132, Guangdong, China; e The State Key Laboratory of Chemical Oncogenomics, Key Laboratory of Chemical Biology, Tsinghua Shenzhen International Graduate School, Tsinghua University, Shenzhen 518055, Guangdong, China; f Institute of Infectious Diseases, Shenzhen Bay Laboratory, Shenzhen 518132, Guangdong, China; g Shenzhen International Institute for Biomedical Research, Shenzhen 518038, Guangdong, China

**Keywords:** Neoantigen, Tumor vaccine, Mass spectrometry, New algorithm

## Abstract

While immune cell therapies have transformed cancer treatment, achieving comparable success in solid tumors remains a significant challenge compared to hematologic malignancies like non-Hodgkin lymphoma (NHL) and multiple myeloma (MM). Over the past four decades, various immunotherapeutic strategies, including tumor vaccines, tumor-infiltrating lymphocyte (TIL) therapies, and T cell receptor (TCR) therapies, have demonstrated clinical efficacy in select solid tumors, suggesting potential advantages over CAR-T and CAR-NK cell therapies in specific contexts. The dynamic nature of the cancer-immunity cycle, characterized by the continuous evolution of tumor-specific neoantigens, enables tumors to evade immune surveillance. This highlights the urgent need for rapid and accurate identification of functional tumor neoantigens to inform the design of personalized tumor vaccines. These vaccines can be based on mRNA, dendritic cells (DCs), or synthetic peptides. In this study, we established a novel platform integrating immunoprecipitation-mass spectrometry (IP-MS) for efficient and direct identification of tumor-specific neoantigen peptides. By combining this approach with our proprietary AI-based prediction algorithm and high-throughput *in vitro* functional validation, we can generate patient-specific neoantigen candidates within six weeks, accelerating personalized tumor vaccine development.

## Highlights

• IP-MS directly identifies *bona fide* personalized neoantigen peptides.
• RNA-seq calibrates the MS data to avoid errors during amino acid *de novo* sequencing.
• A new algorithm prediction is better than netMHCpan4.1.
• Rapid *in vitro* validation ensures neoantigen candidates are functional for tumor vaccine development.

## Graphical abstract

**Figure:**
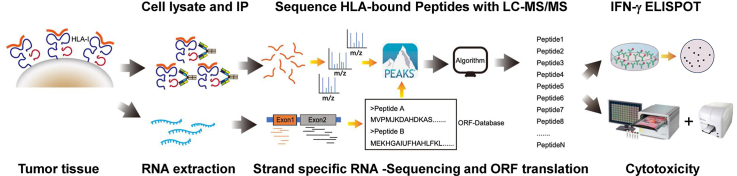

## 1 Introduction

Cancer remains a leading cause of mortality worldwide. Despite advancements such as CAR-T cell therapy and therapeutic antibodies, these treatments exhibit efficacy only in specific cancer types(Miao et al., 2024; Vera et al., 2024). These therapies typically target tumor-associated antigens (TAAs) expressed on the cell membrane surface, activating cytotoxic effector cells either directly or via antibody-dependent cellular cytotoxicity (ADCC)(Guan et al., 2024). However, TAAs often show the antigen density, present a quantitative rather than qualitative difference between tumor and normal cells, leading to “on-target, off-tumor” side effects, which can be toxic(Dharani et al., 2024). In contrast, tumor-specific antigens (TSAs), derived from mutated tumor proteins and processed into 8–12 amino acid peptides (neoantigens), offer a more selective approach. These neoantigens are presented on the cell surface by the major histocompatibility complex (MHC), known as human leukocyte antigen (HLA) in humans (Abdollahi et al., 2024; Janelle et al., 2020), forming a peptide-MHC complex (pMHC) that can trigger specific T cell immune responses through T cell receptor (TCR) engagement(Smith et al., 2015). This pMHC-TCR interaction forms the basis for tumor vaccines, a promising therapeutic modality(Buonaguro & Tagliamonte, 2023; Nguyen et al., 2016).

Tumor vaccines, with a history spanning over 25 years, have generally progressed through three main stages of development: pure or modified peptides, dendritic cell (DC) vaccines, and mRNA vaccines. Early clinical trials utilized synthesized tumor neoantigen peptides to treat patients with metastatic melanoma(Rosenberg et al., 1998). Over the past decade, advances in tumor vaccines have accelerated, paralleling progress in other immunotherapies. Loading neoantigens onto DCs, or engineering DCs to present neoantigens, has become an increasingly adopted strategy(Ding et al., 2021; Lesterhuis et al., 2011). In 2010, Sipuleucel-T (Provenge®), the first DC vaccine, was approved for the treatment of prostate cancer (Borno et al., 2020; Sartor et al., 2020), leading to the registration of hundreds of clinical studies. Concurrently, the widespread adoption of next-generation sequencing (NGS) has facilitated the expansion of neoantigen identification for diverse indications. For instance, researchers reported the discovery of 26 significant gene mutations through whole-xxome sequencing (WES), subsequently screening the KRAS (G12D) mutation and observing tumor regression following re-infusion of TIL cells stimulated by these neoantigens (Tran et al., 2014, 2016).

mRNA tumor vaccines also utilize WES to sequence patient tumor samples and predict the interaction between MHC-I epitopes and potential neoantigens. Subsequently, mRNA loaded with these predicted neoantigens is administered to patients, resulting in observed therapeutic efficacies(Lang et al., 2022; Sahin et al., 2017). This technology was the foundation for BioNTech, which gained global recognition for its COVID-19 mRNA vaccine. Beyond viral mRNA vaccines, BioNTech has advanced three tumor vaccine pipelines with personalized neoantigens into phase II trials, while Moderna has three clinical phase III pipelines(Kopetz et al., 2022; Weber et al., 2024).

NGS currently remains the predominant technique in tumor vaccine development. By comparing transcriptome sequences from patient derived normal and tumor tissues, researchers identify mutated gene sequences within tumor tissue and perform comprehensive analyses to predict potential tumor neoantigens(Karasaki et al., 2017; Peng et al., 2019). However, these candidates are predicted from DNA/RNA sequences, rather than directly from the antigenic peptides (8-12 mers) presented by MHC molecules. Consequently, regardless of whether mutations are point mutations or frameshift mutations, the precise start and end points of these neoantigens cannot be accurately predicted. Although AI is increasingly being leveraged to enhance NGS-based prediction, direct discovery of *bona fide* neoantigens is considered more reliable. Furthermore, the same TSA cannot guarantee the generation of identical neoantigens in the same patient across different time points(Ford et al., 2018; Sharpnack et al., 2022). Therefore, a significant need exists for the rapid and accurate identification of authentic tumor-specific neoantigens.

Recently, immunoprecipitation-mass spectrometry (IP-MS) has emerged as a method for identifying tumor-specific neoantigen sequences, representing authentic peptides eluted from the pMHC complex(Huang et al., 2024; Pak et al., 2021; Sturm et al., 2021). In this study, we enhanced the IP-MS method by integrating RNA-seq to calibrate potential errors arising from MS *de novo* sequencing. Furthermore, our collaborator developed a novel algorithm that yielded divergent prediction results compared to netMHCpan4.1, and these results were subsequently validated through functional experiments. This collaborative approach enriches the research process and leads to more innovative outcomes.

## 2 Results

### 2.1 Advancing tumor-specific neoantigen discovery

**Fig. 1:**
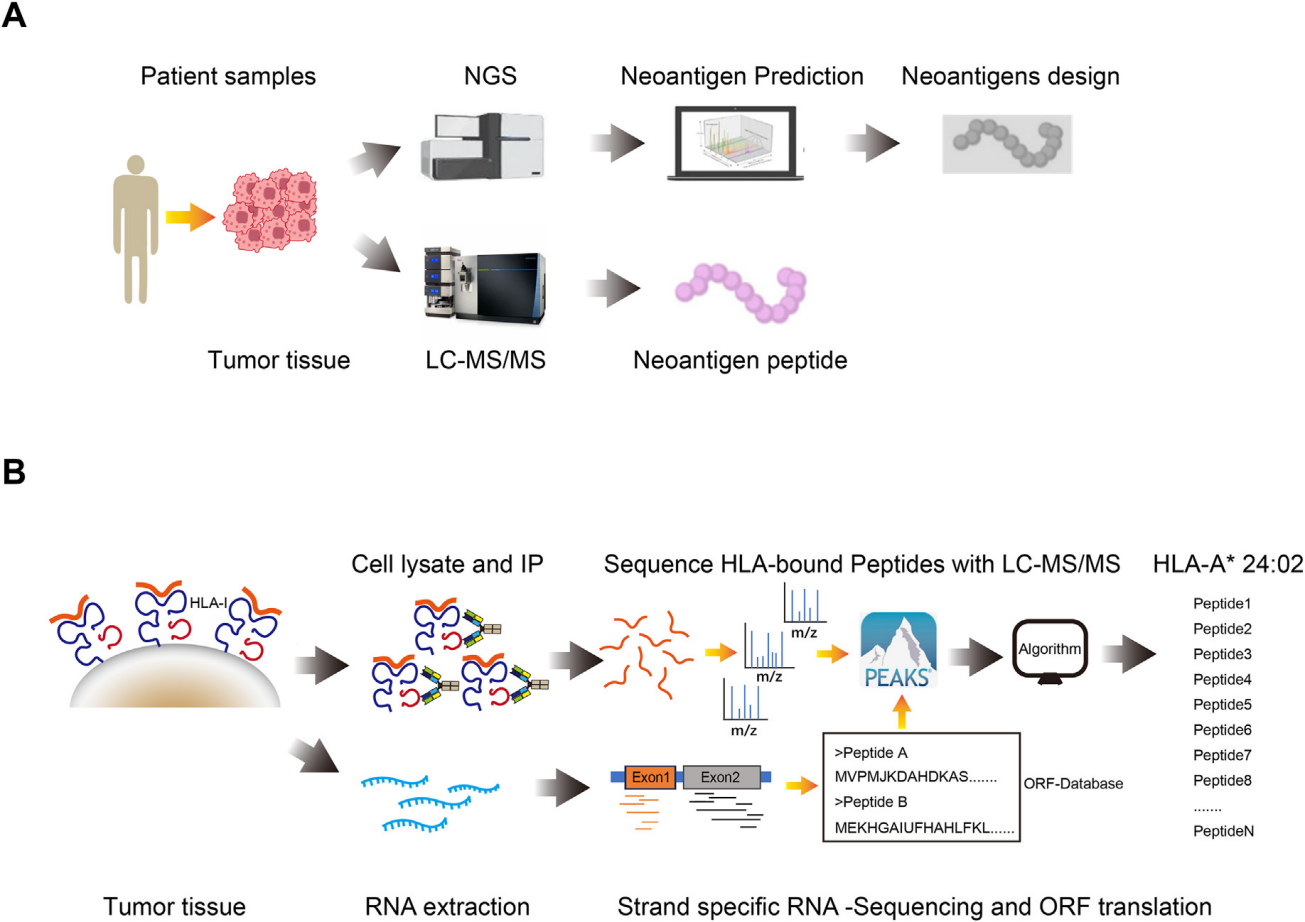
**Comparison between traditional neoantigen identification methods and the technical approach in this study.** (A) Current methods for discovering tumor neoantigens. (B) Schematic diagram of technical methods used in this research.

Neoantigen-based tumor vaccines have shown promise over the past two decades. The conventional approach for personalized tumor neoantigen screening uses DNA/RNA NGS, which translates nucleotide sequences into amino acid sequences to predict potential neoantigen combinations(Ding et al., 2021; Rojas et al., 2023). Although artificial intelligence improves these predictions, it still falls short of directly identifying authentic neoantigen peptides. IP-MS has recently emerged as a method for identifying tumor neoantigens(Huang et al., 2024; Pak et al., 2021; Sturm et al., 2021), often used with algorithms like netMHCpan4.1 for neoantigen scoring (Fig. 1(A)). Given the co-evolution of tumors and the immune system, driven by the cancer-immunity cycle, the most effective neoantigens must be personalized for each patient and can change over time(Mellman et al., 2023). Even within the same patient, rapid and accurate identification of functional tumor neoantigens is, therefore, crucial for optimizing therapeutic windows and maximizing efficacy in treating relapsed and refractory (R/R) cancers.

Because NGS methods do not directly provide peptide information displayed in pMHC complexes, we chose the IP-MS method. We improved the IP-MS approach by integrating RNA-seq data to correct potential errors from MS *de novo* sequencing. Furthermore, our collaborator Dr. Yuzong Chen developed a novel algorithm (details to be published separately), which provides different prediction results compared to netMHCpan4.1 (Fig. 1(B)). This platform has been biochemically validated, tested with tumor cell lines, and analyzed with clinical samples (data not shown). In this study, we describe the technical aspects of this platform using a tumor cell line and its xenograft model.

### 2.2 Immunoprecipitation of pMHC complexes for LC-MS/MS analysis

**Fig. 2:**
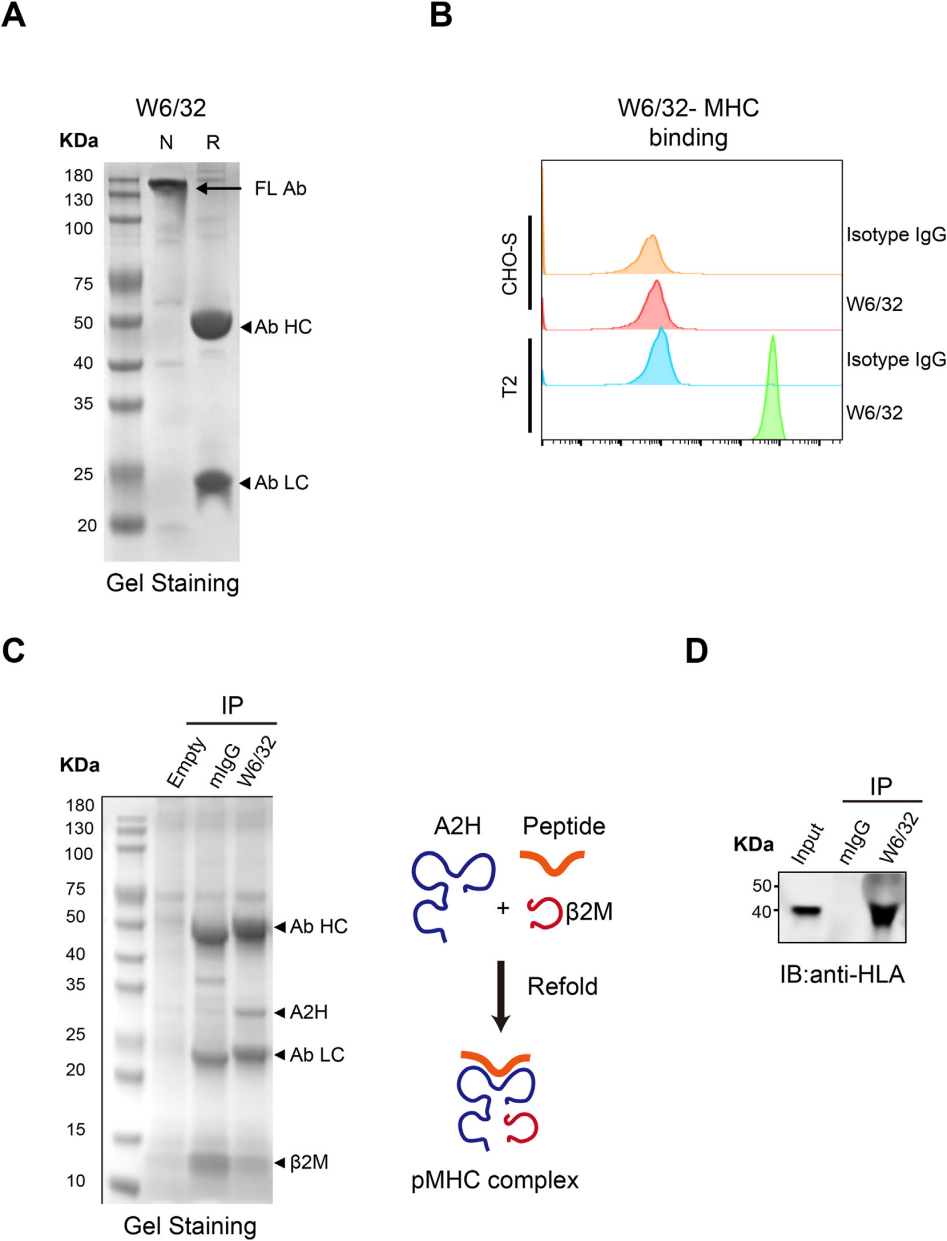
**Immunoprecipitation pMHC complex of Xenograft tumor tissue.** (A) Identify W6/32 antibody purity by SDS-PAGE with non-reducing or reducing loading buffer. N, non-reducing; R, reducing; FL, Full Length; HC, heavy chain; LC, light chain. (B) Quality Controlling the binding affinity of W6/32 with MHC complex using T2 cell line or negative controlled CHO-S cell line. (C) Immunoprecipitant the refold pMHC complex with W6/32. All protein samples were loaded with reduced buffer. The result was presented by Coomassie staining, right panel. HC, heavy chain; LC, light chain. Schematic of pMHC refold in biochemical condition, left panel. (D) Immunoprecipitant the MIA PaCa-2 Xenograft tumor lysate with W6/32. The result was shown by Western Blot.

To identify presented peptides, we performed pMHC - IP-MS on a xenograft tumor sample. To simulate *in vivo* tumor growth, the MIA PaCa-2 cell line was subcutaneously injected into SCID-beige mice. Once tumors reached a volume of 1000 mm^3^, they were harvested and lysed to extract proteins. These proteins were then immunoprecipitated using the W6/32 antibody, which was validated for quality control before immunoprecipitation (Fig. 2(A) and (B)). To confirm the antibody's ability to enrich the pMHC complex under defined biochemical conditions, we expressed the HLA heavy chain A2H (HLA-A2) and the HLA light chain β2M in *E. coli*. The purified A2H, β2M, and a synthesized peptide were then refolded into the pMHC complex (Fig. S1) following established protocols. The results demonstrated that W6/32 successfully immunoprecipitated the pMHC complex, as evidenced by a sharp, clear band for A2H, while the β2M band appeared condensed with nonspecific bands at the bottom (Fig. 2(C)).

**Fig. 3:**
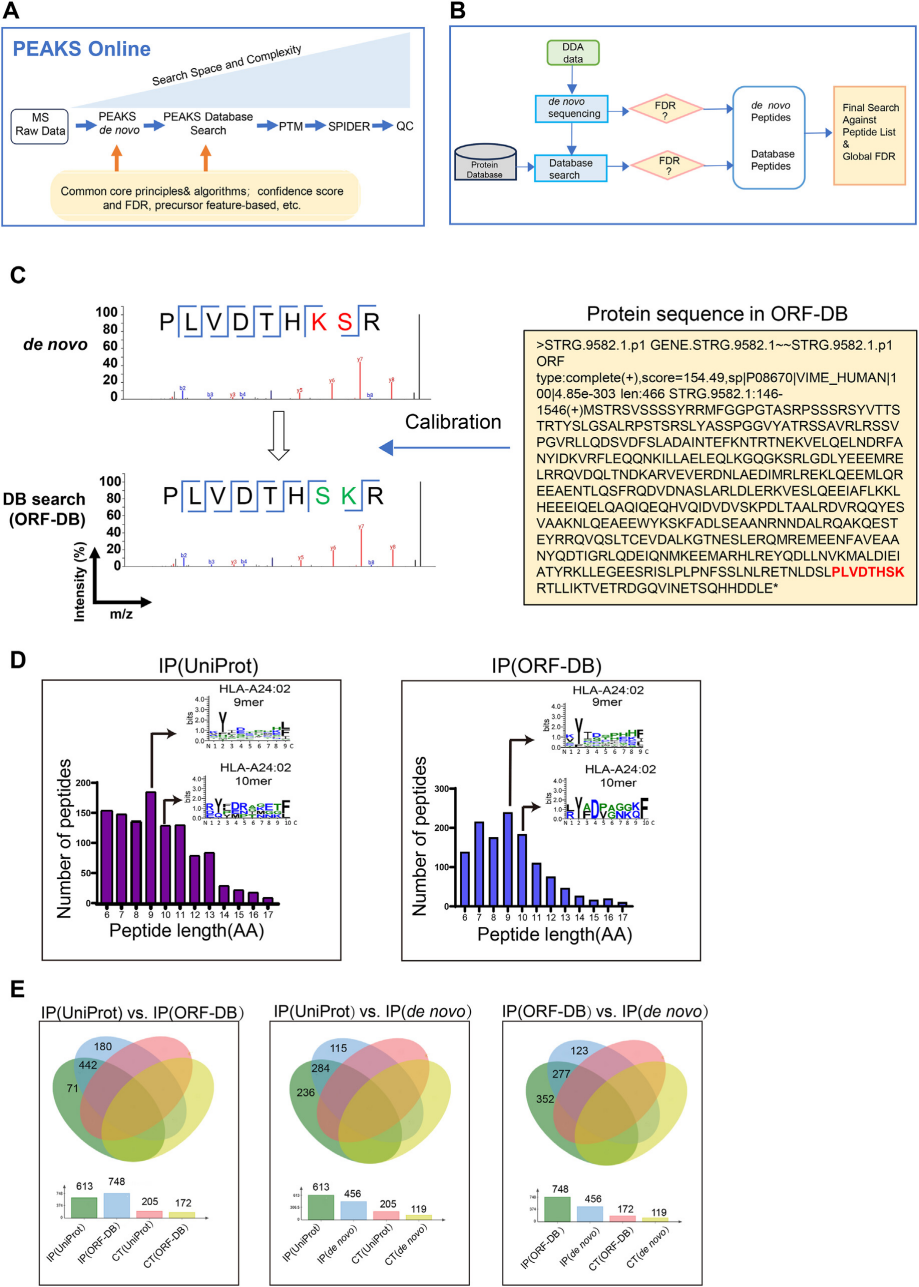
**The analysis of MS result in PEAKS Online.** (A) Overview of data acquisition strategies, analysis approaches, and core algorithms of PEAKS Online. (B) An example of integrated workflow for DDA data analysis. DDA:Data-dependent acquisition. (C) An example of ORF-DB calibrated the error of *de novo* sequencing. The *de novo* sequencing mistake was marked in red while the accurate peptide sequence in ORF-DB was marked in green. ORF-DB: a peptide database of open reading frame (ORF) translations based on RNA-seq data. (D) Peptides length distribution and Sequence logos of IP(UniProt) and IP(ORF-DB). (E) The peptides overlapping of IP(UniProt) vs. IP(ORF-DB), IP(UniProt) vs. IP(*de novo)* and IP(ORF-DB) vs. IP(*de novo*); CT(UniProt)/CT(ORF-DB)/CT(*de novo*) was a control group to filter the non-specific binding peptides; IP: immunoprecipitated with W6/32; CT: immunoprecipitated with mIgG.

Furthermore, W6/32 was used to enrich the pMHC complex from MIA PaCa-2 xenograft tumor lysate, and the results indicated successful precipitation of the pMHC complex (Fig. 2(D)). The eluted peptides were subsequently ultrafiltered and analyzed by LC-MS/MS. PEAKS Online was used to analyze the MS raw data, with the principles of the PEAKS Online workflow and data-dependent acquisition (DDA) analysis detailed in Fig. 3(A) and (B).

### 2.3 Calibrating peptide sequences with RNA-seq and ORF library

Although IP-MS can directly identify tumor neoantigens, rigorous validation is essential. Peptide *de novo* sequencing relies on tandem mass spectrometry to calculate the mass of amino acid residues on the peptide backbone based on the mass difference between two fragment ions. During this process, precursor ions of neoantigens are isolated in the quadrupole and fragmented via collision-induced dissociation with nitrogen gas, producing b- and y-ions. Sufficient fragmentation, indicated by high cleavage coverage along the neoantigen peptide sequence, provides critical information for the *de novo* sequencing algorithm. In our analysis, incomplete peptide fragmentation during mass spectrometry (MS) can lead to ambiguous sequence assignments (Fig. 3(C)). For example, a fragmentation pattern with 87.5% sequence coverage (7 out of 8 possible cleavage sites identified) suggested that a neoantigen sequence could be either “PLVDTHKSR” or “PLVDTHSKR.” This uncertainty, arising from incomplete fragmentation, can introduce errors in *de novo* assembly. To address this, we created a peptide database of open reading frame (ORF) translations based on RNA-seq data, termed ORF-DB. This peptide library was generated through ORF translation (Fig. S2), using ORF-DB as a reference to assemble RNA-seq reads into amino acid sequences for MS analysis. To ensure accuracy, RNA-seq results underwent stringent quality control, achieving an overall alignment rate of 94.69%. This process yielded 14,390 genes, 33,676 transcripts, and 22,875 that matched the reference genome (Table S1–1#). In total, we identified 18,211 single-nucleotide polymorphisms (SNPs) and 1567 insertions/deletions (indels) in the sample (Tables S1–2#). On average, there were 3228 synonymous and 2070 non-synonymous mutations within the coding region (Tables S1–3#). Overall, 1480 mutated genes were identified among the 2070 non-synonymous mutations (Table S2).

### 2.4 Neoantigen identification in MIA PaCa-2 xenograft tumors

The software module PEAKS Online was used to analyze the MS raw data for neoantigen identification via database searching and *de novo* sequencing. For database searching, we used the widely accepted proteomics reference library, UniProt (human, Swiss-Prot). Matching the MS results with the UniProt database identified 1869 peptides, designated IP(UniProt) (Table S3). Following our established workflow, we also aligned the MS results with our ORF-DB, yielding 1526 peptides (designated IP(ORF-DB), Table S4). Analysis indicated that most identified peptides were between 8 and 12 amino acids in length, with 9-mer peptides being the most abundant. Prediction results showed that peptides binding to HLA-A∗24:02 were predominantly 9-mer and 10-mer peptides (Fig. 3(D)). To validate the accuracy of IP(ORF-DB), we compared the overlap between IP(UniProt) and IP(ORF-DB). Using a CAA threshold (CAA% > 0), we obtained 614 peptides of 8–12 amino acids from IP(UniProt) and 748 peptides of the same length from IP(ORF-DB). This comparison revealed 442 overlapping peptides (Fig. 3(E) left, Table S6), indicating high feasibility of the results.

Next, we aimed to validate the accuracy of the peptides identified through *de novo* sequencing (Table S5). Comparing the *de novo* sequencing dataset with the database search results, we found 284 identical peptides between IP(*de novo*) and IP(UniProt), accounting for 62% of the IP(*de novo*) peptides (Fig. 3(E) middle, Table S7). Additionally, there were 277 overlapping peptides when compared with the dataset from ORF-DB, constituting 60% of the IP(*de novo*) peptides (Fig. 3(E) right, Table S8). These findings suggest that errors may have occurred during peptide sequence assembly in the *de novo* process.

Given the significant interest in HLA-A in immunotherapies, we identified the HLA genotype of MIA PaCa-2 as HLA-A∗24:02 (Fig. S3). Through comprehensive data analysis, we focused on the most frequent peptides, specifically those with lengths of 8–12 amino acids. Using both netMHCpan4.1 and our new algorithm, we predicted their binding affinity to HLA-A∗24:02, with 9-mer peptides constituting the largest proportion (Table 1).

**Table 1:** Affinity prediction analysis of netMHCpan4.1 and New algorithm.

Peptide No.	Peptide	CAA(%)	Length	HLA	netMHCpan4.1 Predicted affinity(nM)	BindLevel	New algorithm Predicted affinity(nM)	BindLevel
1	EYPDRIMNTF	50	10	A∗24:02	106.24	SB	157.59	SB
2	FEGFPDKQPR	80	10	A∗24:02	42011.50	NB	33321.40	NB
3	LYADVGGKQF	100	10	A∗24:02	241.58	SB	315.21	SB
4	RYFDPANGKF	80	10	A∗24:02	52.65	SB	307.32	SB
5	YDESGPSIVH	70	10	A∗24:02	45240.08	NB	31383.19	NB
6	AYVHMVTHF	44.4	9	A∗24:02	24.67	SB	37.85	SB
7	EYNSDLHQF	77.8	9	A∗24:02	119.28	SB	331.30	SB
8	KFIDTTSKF	100	9	A∗24:02	70.39	SB	1263.09	WB
9	KYISGPHEL	88.9	9	A∗24:02	51.21	SB	489.38	SB
10	RYIDTHNRV	66.7	9	A∗24:02	102.93	SB	1985.71	WB
11	TYGEIFEKF	77.8	9	A∗24:02	6.46	SB	84.16	SB
12	VYIKHPVSL	100	9	A∗24:02	45.15	SB	107.29	SB
13	VYISEHEHF	77.8	9	A∗24:02	10.33	SB	102.06	SB
14	VYPDGIRHI	55.6	9	A∗24:02	131.16	SB	391.65	SB
15	KYTPPPHHI	66.7	9	A∗24:02	87.68	SB	865.07	SB
16	KYTPDAMLH	77.8	9	A∗24:02	20306.50	NB	3011.98	WB
17	SGPERILSI	66.7	9	A∗24:02	7074.60	NB	4970.61	WB
18	SFVDTRTLL	66.7	9	A∗24:02	3106.86	WB	14600.04	NB
19	LTLGEFLKL	88.9	9	A∗24:02	7160.54	NB	5263.05	WB

SB= strong binding WB=weak binding NB=non binding.

Moreover, the W6/32 antibody binds to all MHC Class I molecules (i.e., HLA-A, HLA-B, and HLA-C), which are crucial for T cell activation. Each individual inherits one set of HLA genes from each parent, leading to a unique combination of HLA alleles. Theoretically, an individual can possess up to 12 different HLA subtypes. Following predictions using netMHCpan4.1 and our new algorithm, we screened out 98 and 108 peptides, respectively (Table S9). In summary, despite bioinformatic predictions, the potential candidate pool remains extensive, highlighting the necessity for high-throughput functional testing.

### 2.5 Comparison of neoantigen identification by IP-MS and MAE

Given that the mild acid elution (MAE) method has been previously used for neoantigen identification(Abdollahi et al., 2024; Sturm et al., 2021), we incorporated this technique alongside our current IP-MS strategy for comparison. Aligning the MAE MS results with the UniProt database, we identified 1980 peptides with lengths of 8–12 amino acids (CAA% > 0) (Table S10–1#). However, only 44 peptides overlapped between the MAE(UniProt) and IP(UniProt) datasets (Fig. S4, Table S10–2#). Among these, 10 peptides were predicted to bind to HLA-A∗24:02 (Fig. S4A), suggesting that the MAE method can offer some insights into specific neoantigens. However, because the peptides identified by the MAE method did not undergo pMHC enrichment, they were more susceptible to contamination by non-target proteins, resulting in a total of 4575 peptides in MAE(UniProt) (Table S10–3#,4#). This increased complexity in screening for true neoantigens ultimately leads to lower confidence in the peptides obtained via the MAE method compared to our IP-MS method.

### 2.6 *In vitro* validation of neoantigen functionality

To validate the functionality of identified neoantigens, we selected 19 peptide sequences (Table 1, Fig. S5) compatible with the HLA-A∗24:02 allele for *in vitro* assays. These candidates were derived from the IP(ORF-DB) dataset, and their binding affinity was predicted using both netMHCpan4.1 and our new algorithm. Two peptides were classified as non-binders (NBs) by both algorithms. The remaining 17 peptides, originating from the 442 peptides common to IP(UniProt) and IP(ORF-DB), were predicted to bind to HLA-A∗24:02. The predicted binding levels of peptides No. 1–15 were similar between the two algorithms; however, the predicted scores for peptides No. 16–19 showed discrepancies.

**Fig. 4:**
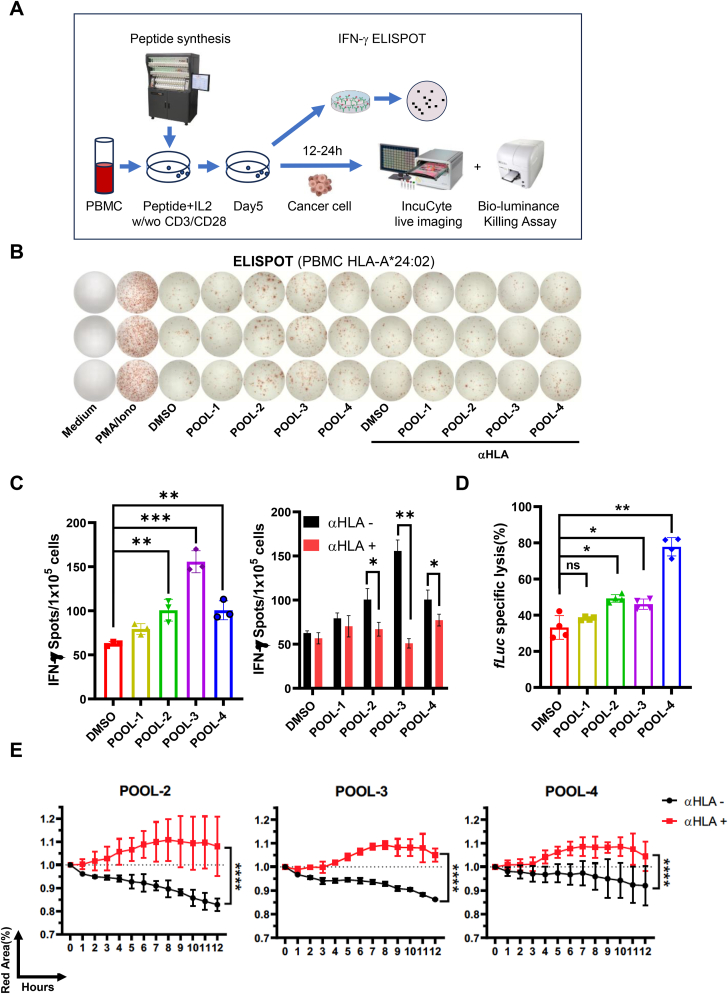
**Neoantigens POOLs activated the Immune responses and cytotoxicity.** (A) Schematic diagram of *in vitro* functional assays of neoantigens, HLA-A∗24:02 PBMCs were used for IFN-γ ELISPOT and cytotoxic assays. w/wo = with or without. (B) PBMCs stimulated by neoantigen pools were used for IFN-γ ELISPOT detection; anti-HLA(W6/32) was used to block T cell recognition; (C) Number of ELISPOTs was calculated. Student two-tailed test. Data represent mean ± SEM from 3 independent replicates (∗*P* < 0.05, ∗∗*P* < 0.01, ∗∗∗*P* < 0.001). αHLA: anti-HLA antibody(W6/32). (D) Detection of neoantigen pools stimulated cytotoxic effects on target cells by using bio-luminance assay. Student two-tailed test. Data represent mean ± SEM from 3 independent replicates (∗*P* < 0.05, ∗∗*P* < 0.01; ns, not significant). fLuc: Firefly luciferase (E) IncuCyte analysis the inhibitory effects of PBMCs against target cells. Anti-HLA(W6/32) was used to block T cell recognition. Student two-tailed test. Data represent mean ± SEM from 3 independent replicates (∗∗∗∗*P* < 0.0001). αHLA: anti-HLA antibody(W6/32). Red area (%) indicates the live cells percentage.

Through this process, we screened candidate antigenic peptides with high predicted affinity for HLA-A∗24:02. To determine whether these peptides genuinely possess immunogenicity, *in vitro* validation was performed (Fig. 4(A)). Given that the natural TCR-neoantigen interaction elicits a mild T cell activation signal, we fine-tuned the *in vitro* experimental design to mimic physiological conditions and demonstrate specific cytotoxicity. To mimic natural T cell signaling, we stimulated peripheral blood mononuclear cells (PBMCs), containing both antigen-presenting cells (APCs) and T cells, with individual peptides. After 5 days of stimulation, the PBMCs were tested using IFN-γ ELISPOT and T cell degranulation assays. To demonstrate specific cytotoxicity *in vitro* following peptide stimulation, anti-CD3/CD28 antibodies were added to activate T cells. Cytotoxicity was assessed using real-time Incucyte live imaging or bioluminescence assays, depending on the experimental needs.

**Fig. 5:**
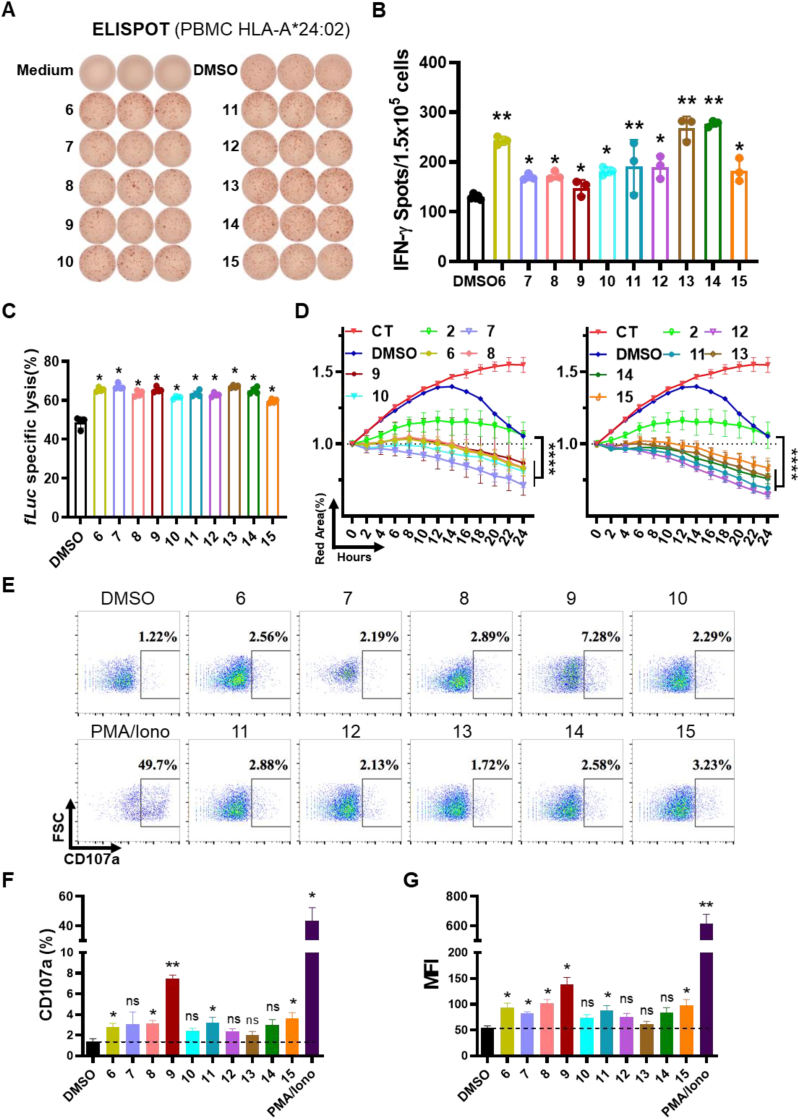
**Screening neoantigens by measuring immune responses and tumor killing effects.** (A) PBMCs induced by No.1-15 peptides were used for IFN-γ ELISPOT detection. (B) Number of ELISPOTs was calculated. Student two-tailed test. Data represent mean ± SEM from 3 independent replicates (∗*P* < 0.05, ∗∗*P* < 0.01). (C) Detection of cytotoxic effects on target cells induced by No.1-15 peptides via using bio-luminance assay. Student two-tailed test. Data represent mean ± SEM from 3 independent replicates (∗*P* < 0.05). fLuc: Firefly luciferase (D) IncuCyte analysis of the inhibitory effects of PBMCs against target cells. Student two-tailed test. Data represent mean ± SEM from 3 independent replicates (∗∗∗∗*P* < 0.0001); the CT group was only without PBMCs. Peptide No.2 was a negative control. The red area (%) indicated live cells percentage. (E–G) CD8+T cells are activated by neoantigens *in vitro*. CD8+T cells were stimulated with peptides or control (DMSO) and the efficacy was evaluated by T cell degranulation. CD107a positive percentage and mean fluorescent intensity were summarized. Data represent mean ± SEM from 3 independent replicates (∗*P* < 0.05, ∗∗*P* < 0.01; ns, not significant). PMA-Iono: PMA-Ionomycin; MFI: Mean fluorescence intensity.

To test our candidates *in vitro*, we first conducted a primary screening of all candidate peptides. Synthesized peptides were used to stimulate allogeneic donor's HLA-A∗24:02 PBMCs, with or without anti-CD3/CD28 antibody. IFN-γ ELISPOT and T cell cytotoxicity assays were then performed. Initially, the peptides were combined into 4–5 peptide pools for rough screening. Subsequently, reactive pools were selected for individual fine screening. Preliminary results indicated that POOL-2 (peptides No.6-10), POOL-3 (peptides No.11-15), and POOL-4 (peptides No.16-19) induced IFN-γ secretion. Unexpectedly, POOL-1 (peptides No.1-5) failed to induce IFN-γ secretion or PBMC cytotoxicity (Fig. 4B–D), even though both algorithms provided strong binding (SB) predictions (Table 1). These results underscore the importance of experimental validation to confirm prediction results before developing tumor vaccines. Furthermore, the killing assay confirmed that POOL-2, POOL-3, and POOL-4 induced PBMCs-mediated cytotoxicity (Fig. 4(E)). The target cell-specific lysis was inhibited by an HLA blocking antibody, consistent with the known ability of the W6/32 antibody to inhibit pMHC-TCR interaction(Matsushita et al., 2016; Yao et al., 2023). Next, to accurately identify the most immunogenic peptide, we stimulated PBMCs with each peptide individually, repeating the IFN-γ ELISPOT assay and evaluating cytotoxicity. Each peptide from POOL-2, 3, and 4 was subjected to individual experiments. The results showed that peptides No. 6–15 induced IFN-γ secretion independently (Fig. 5(A) and (B)) and stimulated PBMCs to lyse cancer cells (Fig. 5(C) and (D)). The T cell degranulation assay also indicated that peptides No. 6–15 activated T cells to varying degrees (Fig. 5(E)–(G), Fig. S6). These results generally aligned with both algorithms' predictions, although some discrepancies were noted.

### 2.7 Comparison of prediction accuracy between algorithms

**Fig. 6:**
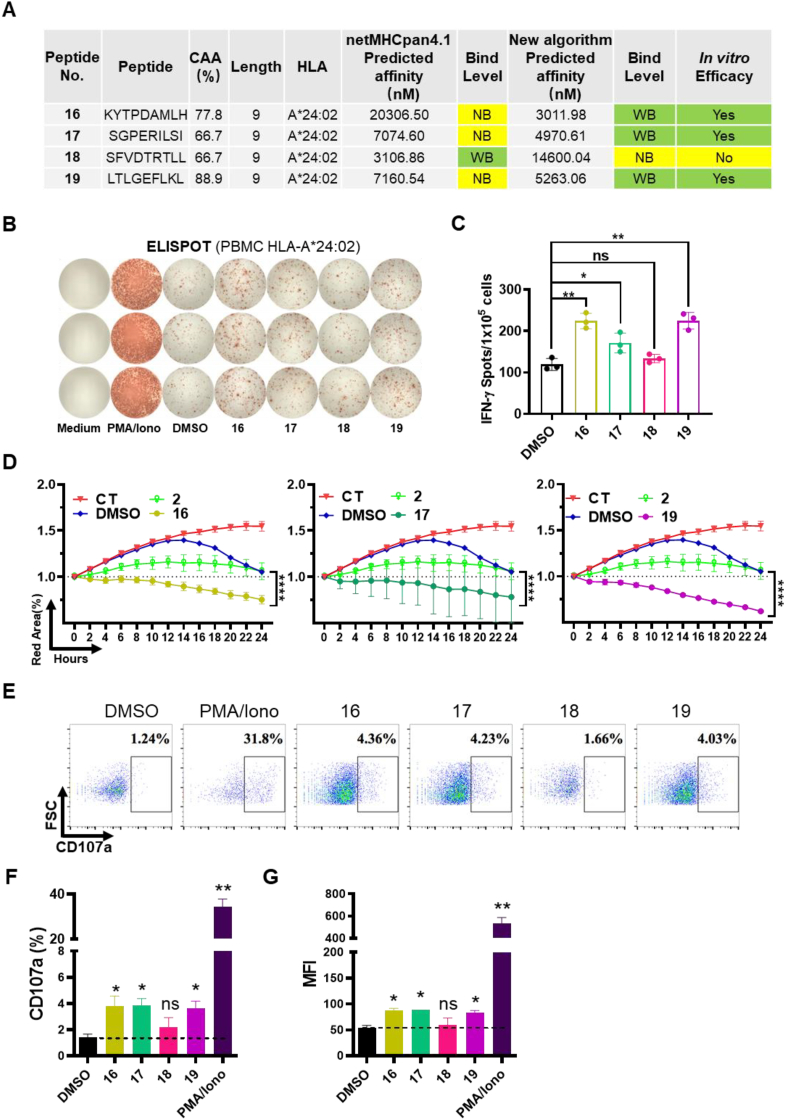
**Measurement of immune responses and tumor killing effects induced byNo.16-19peptides.** (A) Comparison of the differences in affinity prediction results for peptides between the new algorithm and netMHCpan4.1. (B) PBMCs induced by No.16-19 peptides were used for IFN-γ ELISPOT assay. PMA-Iono: PMA-Ionomycin; (C) Number of ELISPOTs was calculated. Student two-tailed test. Data represent mean ± SEM from 3 independent replicates (∗*P* < 0.05, ∗∗*P* < 0.01; ns, not significant). (D) IncuCyte analysis of the inhibitory effects of PBMCs against target cells. Student two-tailed test. Data represent mean ± SEM from 3 independent replicates (∗∗∗∗*P* < 0.0001); the CT group was only MIA PaCa-2 without PBMCs. Peptide No.2 was a negative control. (E)–(G) CD8+T cells activated by neoantigens *in vitro*. CD8+T cells were stimulated with peptides or control (DMSO) and the efficacy was evaluated by T cell degranulation. CD107a positive percentage and mean fluorescent intensity were summarized. MFI: Mean fluorescence intensity. Data represent mean ± SEM from 3 independent replicates (∗*P* < 0.05, ∗∗*P* < 0.01; ns, not significant).

This study employed netMHCpan4.1, an AI algorithm based on a neural network, alongside our innovative new algorithm developed by Dr. Yuzong Chen (publication forthcoming). This new algorithm is a deep learning-based predictive model integrating biological knowledge and trained on quantitative measurements of peptide-MHC binding affinity (Shen et al., 2021, 2022). Given that both neural networks and deep learning are major algorithms for AI development, we compared their differences in Table S11. Our experimental findings suggest that the predictions made by the two algorithms show a considerable degree of agreement. However, we observed inconsistencies, particularly in the peptide predictions No. 16–19 (Fig. 6(A)). To ascertain which algorithm yielded more accurate results, we conducted further *in vitro* evaluation experiments. The results showed that peptides predicted as non-binders (NBs) by netMHCpan4.1, specifically No. 16, No. 17, and No. 19, stimulated immune cells to produce IFN-γ and triggered immune cells to eliminate cancer cells. Conversely, peptide No. 18, predicted as weak binding (WB) by netMHCpan4.1, did not exhibit these functionalities (Fig. 6(B)–(G)). These findings indicate that our new algorithm's predictions surpass those of netMHCpan4.1 in terms of accuracy.

### 2.8 TCR activity towards the validated neoantigen

**Fig. 7:**
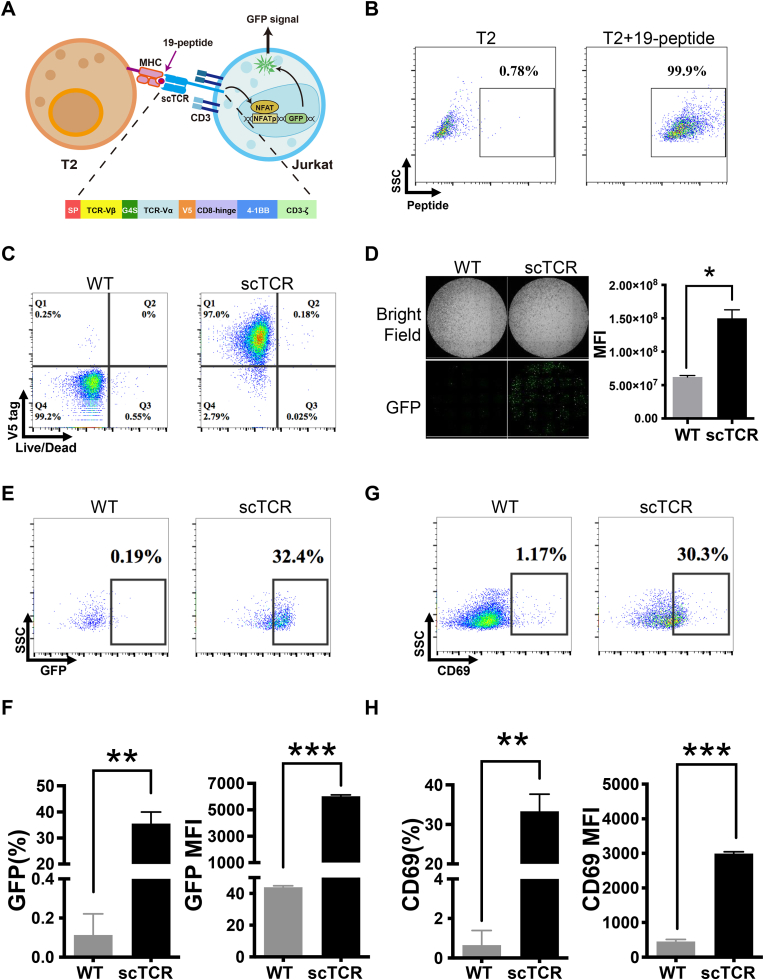
**No.19 peptide loaded pMHC triggers T cell activation via the specific TCR interaction.** (A) Schematic of scTCR Jurkat-NFAT-GFP activated upon binding with T2 loaded with No.19 peptide. (B) 10 μg/mL FITC conjugated No.19 Peptide was loaded onto empty T2 cells for 2 h at 37 °C, T2 cells were then rinsed twice and checked via FACS and controlled by T2 cells without any peptide loading. (C) Jurkat NFAT-GFP were stably transduced with the lentivirus containing scTCR-V5-BBZ. The positive transduced cells were polyclonal sorted by Flow cytometer. The sorted cells were maintained and the scTCR expression was checked before co-culture. (D) 24 h post of the co-culture of No.19 peptide loaded T2 cells and the Jurkat NFAT-GFP cells. High-content imaging analysis at 10 × magnification was applied to evaluate the NFAT-GFP reporter signals (Left). Besides, the Mean fluorescence intensity (MFI) of GFP (right) was quantified by the imaging software. Data represent mean ± SEM from 3 independent replicates (∗*P* < 0.05). (E)–(H) After High-content imaging analysis, the co-culture cells were harvested and stained with viability dye, CD3, and CD69 antibodies. Flow cytometry was applied to compare the NFAT-GFP reporter signals ((E)–(F)) as well as the CD69 expression stands for the T cell activation ((G)-(H)). Data represent mean ± SEM from 3 independent replicates (∗∗*P* < 0.01, ∗∗∗*P* < 0.001).

Among the neoantigens screened, an ideal candidate would be functional for developing tumor vaccines or TCR-based therapeutics. We identified peptide No. 19, derived from Survivin, a promising target with an existing clinical-phase TCR drug from AbbVie(Chervin, & et al., 2021). ABBV-184 is a TCR-CD3 engager protein drug targeting the same peptide as No. 19 in our list(Chervin et al., 2023; Peterlin et al., 2024). To evaluate the binding between the MHC complex loaded with peptide No. 19 and the TCR, as well as the consequent T cell signaling, we performed an *in vitro* T cell-specific activation assay (Fig. 7(A)). First, T2 cells, which express MHC molecules without any pre-loaded peptides, were loaded with FITC-conjugated peptide No. 19. Peptide loading was verified by flow cytometry (Fig. 7(B)). Second, we established a Jurkat NFAT-GFP reporter cell line (Fig. S7). Furthermore, we cloned the single-chain TCR sequence (scTCR) from ABBV-184 into a lentiviral-based 2nd generation CAR backbone and transduced this scTCR into the Jurkat NFAT-GFP cell line to establish a stable scTCR reporter cell line (Fig. 7(C)). Co-culturing peptide No. 19-loaded T2 cells with either wild-type or scTCR-stable Jurkat NFAT-GFP cells, we observed that scTCR Jurkat cells significantly boosted NFAT-GFP reporter expression upon contact with peptide No. 19-loaded T2 cells, consistent with previous reports (Fig. 7(D)). Finally, we harvested the co-cultured cells, stained them with a T cell marker (CD3) and a T cell activation marker (CD69), and analyzed them via flow cytometry alongside the NFAT-GFP reporter (Fig. 7(E)–(H)). These results demonstrate that peptide No. 19, identified using our platform, can react with its targeted TCR and induce a robust T cell activation signal.

## 3 Discussion

Neoantigen discovery and identification, particularly of tumor-specific antigens resulting from cancer cell mutations, offers significant potential for improving cancer treatment and advancing tumor immunotherapy. Given the critical role of individual genetic backgrounds in determining neoantigen effectiveness, personalized approaches to tumor vaccines are essential.

Detecting neoantigen expression in tumor tissues enables the screening of suitable antigens for vaccine design. This enhances therapeutic efficacy by ensuring vaccines elicit a robust immune response against the unique mutations present in an individual's tumor. Rapid neoantigen identification can improve treatment outcomes, reduce recurrence rates, and propel tumor immunotherapy forward.

Despite advancements in NGS technology, directly identifying neoantigen peptide sequences remains challenging due to the complexities of peptide splicing and post-translational modifications that NGS alone cannot resolve. To address these limitations, a novel approach has been introduced: constructing a tumor tissue-specific peptide database by translating open reading frames (ORFs) derived from RNA-seq results. This ORF-DB more accurately reflects the tumor's characteristics compared to traditional databases like UniProt, enhancing neoantigen sequence identification precision. Integrating MS results can further aid in obtaining neoantigen sequence information and predicting their binding affinity to HLA alleles. Enrichment-based approaches significantly improve neoantigen identification accuracy, as traditional methods like MAE may yield lower authenticity due to potential contamination with non-target proteins. A collaborative approach enriches the research process and leads to more innovative and inclusive outcomes.

This research aims to develop a method for the rapid and precise identification of effective neoantigens from tumor tissues. Initially, a peptide database specific to the tumor tissue was constructed by translating ORFs derived from RNA-seq results on the tumor tissue. Although the traditional UniProt database (human, Swiss-Prot) contains a more significant number of proteins and can provide more peptide information, the ORF-DB developed in this study more accurately reflects the characteristics of the tumor tissue than the UniProt database. By comparing these two database search methods, information about *bona fide* neoantigens can be accurately obtained. *De novo* sequencing serves as a fundamental analytical function of PEAKS. However, during the experimental process, it was found that errors can occur during the assembly process due to incomplete fragmentation of peptide segments. Such errors can be rectified through a database search with ORF-DB. This approach has the potential to enhance the precision of neoantigen sequence identification.

The RNA-seq result indicates the presence of 1480 mutated genes in the MIA PaCa-2 cell line. However, the neoantigens produced by these mutations were not identified, indicating that the proteins encoded by the mutated genes may not necessarily be presented by HLA. Previous studies have also indicated that the mutation burden in pancreatic cancer is relatively low, which aligns with these findings(Rojas et al., 2023).

After analyzing the MS results to obtain neoantigen sequence information, these results were combined with the sequencing outcomes of HLA typing. The binding affinities of the neoantigens for HLA-A∗24:02 were predicted and scored using netMHCpan4.1 and a proprietary algorithm. The scores produced by the developed algorithm were mostly consistent with those from netMHCpan4.1.

Additional studies are necessary to verify the potential applications of these neoantigens. It is important to understand their biological functions in detail. For example, *in vitro* assays have demonstrated that the representative neoantigens are immunogenic. Even some peptides are predicted to have strong binding to HLA-A∗24:02 but are unable to induce an immune response in PBMCs.

The findings suggested that the two algorithms' predictions are almost consistent. Nevertheless, discrepancies were noted in some neoantigens. To ascertain the reliability of each algorithm's predictions, supplementary *in vitro* evaluations were carried out. These experiments showed that *in vitro* assays are more consistent with the prediction from the new algorithm. These results underscore the superior accuracy of the new algorithm compared to netMHCpan4.1 in predicting peptide binding affinity.

In summary, the rapid and precise identification of effective neoantigens from tumor tissues is crucial for enhancing cancer treatment and advancing tumor immunotherapy. The development of tailored tumor vaccines based on individual genetic profiles, coupled with innovative methodologies for neoantigen discovery, represents a significant stride toward personalized cancer therapies. This research not only contributes to the understanding of neoantigen biology but also lays the groundwork for future advancements in cancer immunotherapy, ultimately aiming to improve patient outcomes and reduce the burden of cancer recurrence.

By establishing a robust workflow for neoantigen discovery and validation, researchers can expedite the transition from identification to pre-clinical functional evaluation, marking a significant milestone in the development of effective cancer vaccines. In business research, incorporating psychological concepts like Theory of Mind (ToM) into management theories exemplifies interdisciplinary synergy, fostering more robust and versatile research frameworks. ToM enables researchers to construct arguments that are attuned to the mental states and motivations of their audience, ensuring that arguments are both persuasive and empathetic.

## 4 Methods

### 4.1 Cells

MIA PaCa-2: The MIA PaCa-2 cell line (American Type Culture Collection [ATCC], Manassas, VA, USA) was cultured in DMEM supplemented with 10% FBS and 1% antibiotics at 37 °C in a 5% CO_2_ atmosphere. Cells were passaged every 2–3 days at a sub-cultivation ratio of 1:3 using trypsinization and were assumed to have undergone 20 passages.

293T: 293T cells, provided by cell stock of Shenzhen Bay Laboratory, were cultured in DMEM supplemented with 10% FBS and 1% antibiotics at 37 °C in a 5% CO_2_ atmosphere.

W6/32 Hybridoma: The W6/32 hybridoma cell line (ATCC, Manassas, VA, USA) was cultured in DMEM supplemented with 10% FBS and 1% antibiotics at 37 °C in a 5% CO_2_ atmosphere. Cells were maintained in triangular culture bottles and passaged every 4–5 days at a sub-cultivation ratio of 1:3. Each cell line was assumed to have undergone 5 passages.

T2: The T2 cell line (ATCC, Manassas, VA, USA) was cultured in RPMI 1640 supplemented with 10% FBS and 1% antibiotics at 37 °C in a 5% CO_2_ atmosphere. Cells were passaged every 2–3 days at a sub-cultivation ratio of 1:3.

CHO-S: CHO-S cells, provided by cell stock of Shenzhen Bay Laboratory, were cultured in DMEM supplemented with 10% FBS and 1% antibiotics at 37 °C in a 5% CO_2_ atmosphere.

Jurkat: Jurkat cells, provided by cell stock of Shenzhen Bay Laboratory, were cultured in RPMI 1640 supplemented with 10% FBS and 1% antibiotics at 37 °C in a 5% CO_2_ atmosphere.

All cell lines were preserved in liquid nitrogen using a mixture of 95% FBS and 5% DMSO.

hPBMC: Human peripheral blood mononuclear cells (hPBMC) were obtained from human peripheral blood provided by Taicang First People's Hospital and hPBMC isolation service provided by Milestone Biotechnologies. hPBMC were preserved in liquid nitrogen using STEMCELL Technologies' CryoStor® CS10 freezing buffer.

### 4.2 Lentiviral packaging

HEK293T cells were utilized as packaging cells for lentivirus production via transfection. Briefly, 1 × 10^7^ 293T cells were seeded onto 10 cm plate and cultured to 80% confluence. A mixture of 60 μg of PEI (Polysciences, 24765-1) and 24 μg of plasmid DNA (pSpAX2: pMD2.G: target vector at a ratio of 5:7:12) was prepared in 0.5 mL Opti-MEM (ThermoFisher, 11058021) by incubating each component separately for 5 min, followed by combining them for 20 min before transfection. Eight hours post-transfection, the medium was replaced with fresh, antibiotic-free medium supplemented with 2% FBS. Supernatants containing lentivirus were collected at 48 and 72 h post-transfection by low-speed centrifugation and filtered through a 0.45 μm syringe filter (Millipore, SLHVR33RB). The resulting lentivirus supernatant was either used immediately, concentrated by centrifugation at 20,000*g* for 2 h, or snap-frozen in liquid nitrogen and stored at −80 °C.

### 4.3 Generation of MIA PaCa-2 stable cell line

MIA PaCa-2 cells were seeded 24 h before transduction. Lentivirus expressing either luciferase or mKate was diluted in DMEM supplemented with 1 μg/mL polybrene. The existing cell culture medium was removed, and the lentivirus/polybrene mixture was directly applied to the cells, followed by incubation for 24 h at 37 °C. Subsequently, the medium was replaced with fresh culture medium. At 48 h post-transduction, puromycin (1 μg/mL) was added to the medium for selection of successfully transduced cells. Luciferase substrate assays were used to quantify luciferase expression in the MIA PaCa-2-luciferase cell line. The expression of red fluorescent protein (mKate) in the MIA PaCa-2-mKate cell line was validated using IncuCyte imaging.

### 4.4 Generation of Jurkat NFAT-GFP reporter cell line

NFAT-GFP lentiviral plasmid was generously provided by Dr. Chen Yu from Shenzhen Bay Laboratory. To generate a stable reporter cell line, Jurkat cells were infected with lentivirus encoding NFAT-GFP. Following lentiviral transduction, single-cell clones were sorted into U-bottom 96-well plates and cultured in RPMI 1640 supplemented with 20% FBS, 1 μg/mL puromycin, and 1% antibiotics. After clonal expansion, each clone was passaged at a 1:2 split ratio. One aliquot of each clone was either untreated or treated with PMA-Ionomycin (ThermoFisher, 00-4975-03). GFP reporter signals were then evaluated via FACS analysis. Positive clones were identified, recorded, and the corresponding aliquot was selected for further experiments.

### 4.5 Generation of Jurkat-scTCR stable cell line

The TCR sequence of ABBV-184 that targets No.19 peptide (CHERVIN, & ET AL., 2021) was synthesized and cloned into a lentiviral vector as a single-chain TCR (scTCR) fused with a V5 tag and the transmembrane and intracellular signaling domains of a second-generation chimeric antigen receptor (CAR). Jurkat NFAT-GFP reporter cells were then transduced with the packaged lentivirus. At 48 h post-transduction, transduction efficiency was assessed by flow cytometry (FACS) via V5 tag staining (ThermoFisher, 12-6796-42). V5-positive polyclonal cells were subsequently sorted to establish a stable cell line.

### 4.6 Jurkat-NFAT-GFP activation assay

Wild-type Jurkat NFAT-GFP cells or Jurkat NFAT-GFP cells stably expressing scTCR were co-cultured with peptide-loaded T2 cells at a 1:1 ratio and incubated for 24 h. NFAT-GFP reporter activity was then quantified using a High-Content Imaging System (PerkinElmer, Opera Phenix Plus) and analyzed with Harmony software. Following co-culture, cells were harvested and stained with a viability dye (Tonbo Bio, 13–0870), anti-CD3 (ThermoFisher, 25-0037-42), and anti-CD69 (Biolegend, 310922) antibodies to assess T cell activation. CD69 expression (a marker of T cell activation) and GFP expression (NFAT reporter activity) were quantitatively analyzed by flow cytometry.

### 4.7 Xenograft model

Female CB17.B6-*Prkdc*^*scid*^*Lyst*^*bg*^/Crl mice (4–6 weeks old, from Beijing Vital River Laboratory Animal Technology Co., Ltd) were used for *in vivo* tumorigenicity studies. Each mouse received a single subcutaneous injection (200 μL) into the right flank, consisting of 1 × 10^7^ MIA PaCa-2 cells suspended in PBS and Matrigel. Injection sites were monitored until palpable tumors were established. Once tumors reached the predetermined size, they were excised for subsequent analysis. All animal studies were conducted following the guidelines and regulations of the Regional Ethics Committee for Animal Experiments at Shenzhen Bay Laboratory. Mice were maintained under standard housing conditions with a 12-h light/dark cycle and ad libitum access to food and water.

### 4.8 Production of W6/32 antibody

8–10 weeks old Balb/c mice from the same consistent lineage were utilized for monoclonal antibody production. To enhance hybridoma cell engraftment, mice were pre-sensitized with an intraperitoneal injection of mineral oil one week before hybridoma cell inoculation. Subsequently, 1 × 10^6^ hybridoma cells were injected into the peritoneal cavity of each mouse. Approximately 7 days post-injection, ascites fluid was harvested. The W6/32 antibody was then purified from the ascites fluid using Protein A Sepharose affinity chromatography. Following elution, the buffer was exchanged for PBS via ultrafiltration.

### 4.9 W6/32 and cell binding

T2 and CHO-S cells were cultured in their respective optimized media. Upon reaching the desired confluency, cells were harvested and centrifuged at 300*g* for 5 min to remove the supernatant. To eliminate residual culture medium components, cells were resuspended in PBS and washed twice. The W6/32 antibody was diluted in PBS to the appropriate working concentration. The diluted W6/32 antibody was added to the cell suspension, with an isotype-matched mouse IgG (mIgG) as a negative control. The mixture was incubated at room temperature for 30 min, unbound primary antibody was rinsed, and secondary antibody (CST, 4410S) was then applied to incubate at room temperature in dark for an additional 30 min. Cells were washed 2–3 times with PBS and were resuspended in PBS at a concentration of 1 × 10^6^ cells/mL and transferred to sample tubes for flow cytometry analysis.

### 4.10 Western Blot

Equal amounts of protein were separated by SDS-PAGE using polyacrylamide gels with a concentration gradient ranging from 10 to 15%. Following electrophoresis, proteins were transferred to a PVDF membrane, which was then blocked with 5% non-fat dry milk. The membrane was subsequently incubated with the following primary antibody: anti-HLA (HC10, ThermoFisher, MUB2037P) at a 1:1000 dilution. After washing, the membrane was incubated with anti-HRP-conjugated secondary antibodies (CST, 7074 or 7076) at a 1:5000 dilution. Protein bands were visualized using enhanced chemiluminescence (ECL) assays (Beyotime, P0018S). Images were cropped to display the molecular weight marker proteins (kDa).

### 4.11 HLA genotyping

HLA genotyping of the MIA PaCa-2 cell line was performed by BGI. Genomic DNA was extracted, and HLA genes were amplified via PCR using sequence-specific primers. The amplified products were then purified and prepared for sequencing. Sequencing was conducted using the 3730xl DNA Analyzer (Applied Biosystems). The resulting sequencing data were analyzed using specialized software to determine the HLA genotype.

### 4.12 RNA-seq and ORF translation

Total RNA was extracted using RNAiso (Takara Bio, 9019). Poly(A) enrichment or ribosomal RNA depletion using specific probes was employed to remove ribosomal RNA. Strand-specific RNA-seq libraries were constructed, selecting for insert fragment sizes between 350 and 450 base pairs. Sequencing was performed on the Illumina NovaSeq™ X Plus platform using paired-end 150 bp reads to generate deep sequencing data. Reads were aligned to the hg38 reference genome (Gencode V38) using Hisat2 V2.2.1 with the following parameters: rna-strandness RF -dta -no-mixed -no-unal. Transcript assembly was performed using Stringtie 2.2.2 (parameter -rf) with reference annotation. ORFs and coding regions were predicted from the assembled GTF output using TransDecoder v5.7.1, and these predicted coding regions were subsequently mapped back to the genome.

### 4.13 pMHC enrichment by immunoprecipitation

For pMHC isolation, MIA PaCa-2 xenograft tumor tissue (100 mg wet weight) was minced and lysed in 500 μL of CHAPS buffer supplemented with protease inhibitors (ThermoFisher, 87785). pMHC complexes were immunoprecipitated using the W6/32 antibody conjugated to Protein A/G magnetic beads (MCE, HY-K0202). Peptides were eluted with 10% acetic acid and subsequently filtered using a 3 kDa molecular weight cut-off ultrafiltration device (Millipore, UFC5003) to retain MHC-I heavy chain (A2H), β2M light chain, and the W6/32 antibody. Peptide extracts were then desalted using C18-ZipTips (Millipore, ZTC18S) and concentrated by vacuum centrifugation. Before mass spectrometry analysis, the peptides were resuspended in 0.1% formic acid (FA) (ThermoFisher, A117-50).

### 4.14 Mild acid elution (MAE)

MIA PaCa-2 Xenograft tumor sample (wet weight 50 mg) was cut into small pieces and washed three times with PBS at 200 g for 10 min centrifugation. Tumor sample was put into MAE buffer (10% acetic acid) for 1 min. The MAE buffer was filtered on a 3 kDa molecular weight cut-off ultrafiltration tube (Millipore, UFC5003) to retain MHC-I A2H heavy chain, β2M light chain, and W6/32 antibody. Peptide extracts were desalted by C18-ZipTips and dried using a Speed-Vac. Before MS analysis, the peptides were resuspended in 0.1% FA.

### 4.15 LC-MS/MS analysis

LC-MS/MS analysis was performed on an Easy nLC 1200 (ThermoFisher, Bremen, Germany) coupled to an Orbitrap Fusion Lumos equipped with a nanospray flex ion source (ThermoFisher, Bremen, Germany). The peptides were dissolved in water with 0.1% FA and separated on a commercial RP-HPLC pre-column (75 μm × 2 cm) (ThermoFisher, 164535) and RP-HPLC analytical column (75 μm × 25 cm) (ThermoFisher, 164941), both packed with 2 μm C18 beads. The peptides were eluted over a 90 min segmented gradient. The Orbitrap Fusion Lumos acquired data in a data-dependent manner, alternating between full-scan MS and MS2 scans. Isolated precursor ions were sequentially fragmented in a 3 s cycle. Dynamic exclusion was set to 30 s, and precursors with charge states were isolated for MS/MS experiments.

### 4.16 MS data processing and database searching

Qualitative and quantitative analysis of mass spectrometry raw data was performed using a multi-stage workflow in PEAKS Online 10 (Bioinformatics Solutions Inc., Waterloo, Canada). Initially, the data were searched against the human UniProt database. This was followed by a *de novo* peptidome sequencing search. Precursor and fragment mass error tolerances were set to 10 ppm and 0.02 Da, respectively. Methionine oxidation (+15.9949 Da) was included as a variable modification. All results were filtered to a 1% false discovery rate (FDR). For *de novo* sequencing, only peptides with a *de novo* score of 70% or higher were retained for subsequent analysis.

### 4.17 Synthetic peptides

Peptides were synthesized using solid-phase peptide synthesis (SPPS) with Fmoc chemistry, proceeding from the C-terminus to the N-terminus. The synthesis employed a resin support, Fmoc-protected amino acids, and appropriate peptide condensation reagents. Following synthesis, peptides were cleaved from the resin and purified by reversed-phase chromatography on a C18 column, using a gradient elution of acetonitrile and water containing 0.05% trifluoroacetic acid. Peptide synthesis was performed using a Symphony X Polypeptide Synthesizer at the Translation Innovation Center of Shenzhen Bay Laboratory or by Genscript. Synthesized peptides were subsequently dissolved in DMSO for downstream assays.

### 4.18 T cell cytotoxicity

Untreated 24-well plates (NEST, 702001) were coated with anti-CD3/CD28 antibodies (1 μg/mL, Biolegend, 317347/302943). Peripheral blood mononuclear cells (PBMCs), cultured in a medium consisting of 45% Click's medium, 45% RPMI 1640, 10% FBS, 1% antibiotics, and 20 Units/mL IL-2, were seeded at a density of 1 × 10^6^ cells per well. PBMCs were stimulated with the indicated peptide(s) or DMSO. Single peptide treatments were performed at a concentration of 10 μg/mL. For mixed peptides treatments, a pool of 4 or 5 peptides was used at a concentration of 2 μg/mL per peptide. An equivalent volume of DMSO was used as a negative control. Five days post-stimulation, PBMCs were harvested and co-cultured overnight with MIA PaCa-2 cells stably expressing either firefly luciferase (fLuc) or mKate at an effector-to-target (E:T) ratio of 20:1. Target cells without any treatment served as the spontaneous death control, while target cells lysed with NP-40 lysis buffer represented the maximal killing control. Bioluminescence-based cytotoxicity was quantified using a BioTek Synergy Neo2 plate reader, and specific lysis was calculated using the following equation: Specific lysis (%) = 100 × (Spontaneous death RLU - Test RLU)/(Spontaneous death RLU - Maximal killing RLU). In parallel, IncuCyte-based cytotoxicity was monitored in real-time by quantitative measurement of live, mKate + tumor cells. Values were normalized to the *t* = 0-h measurement.

### 4.19 ELISPOT

IFN-γ ELISPOT assays were. PBMCs were seeded at a density of 1 × 10^6^ cells per well. PBMCs were stimulated with the indicated peptide(s), PMA-Ionomycin (positive control), or DMSO (negative control). Five days post-stimulation, stimulated PBMCs were. IFN-γ spots were quantified using a CTL S6 Ultra ELISPOT reader.

IFN-γ ELISPOT assays were performed using an ELISPOT kit (BD Biosciences, 551849). 1 × 10^6^ PBMCs were seeded per well. PBMCs were treated with the indicated peptide(s), PMA-Ionomycin, or DMSO. 5 days post-treatment, stimulated PBMCs were performed using an ELISPOT kit (BD Biosciences, 551849). IFN-γ spots were counted using CTL S6 Ultra.

### 4.20 T cell degranulation

PBMCs were seeded at a density of 1 × 10^6^ cells per well. PBMCs were stimulated with the indicated peptide(s), PMA-Ionomycin (positive control), or DMSO (negative control). Seven days post-stimulation, PBMCs and target cells were co-cultured. Concurrently, APC-conjugated anti-CD107a antibody (Biolegend, 301102) was added to each well at 2.5 μL per test. After 1 h, Monensin (ThermoFisher, 00-4505-51) was added, and co-culture was continued for an additional 5.5 h. CD8-FITC antibody (Biolegend, 301505) was added for the last 30 min to facilitate T cell gating. CD107a staining was then quantified by flow cytometry.

## CRediT authorship contribution statement

**Huajian Tian:** Writing – original draft, Visualization, Validation, Formal analysis, Data curation. **Guifei Li:** Writing – review & editing, Validation, Formal analysis, Data curation. **Cookson K.C. Chiu:** Validation, Software, Formal analysis, Data curation. **E. Li:** Validation, Data curation. **Yuzong Chen:** Resources, Software. **Ting Zhu:** Investigation, Software. **Min Hu:** Investigation, Software. **Yanjie Wang:** Investigation, Software. **Suping Wen:** Formal analysis, Investigation. **Jiajia Li:** Resources. **Shuangxue Luo:** Resources. **Zhicheng Chen:** Resources. **Huimei Zeng:** Resources. **Nan Zheng:** Resources. **Jinyong Wang:** Resources. **Weijun Shen:** Writing – review & editing, Project administration, Funding acquisition. **Xi Kang:** Writing – review & editing, Validation, Supervision, Resources, Project administration, Methodology, Investigation, Formal analysis, Conceptualization.

## Conflict of interest statement

The authors declare that there are no conflicts of interest related to this manuscript.

## Figures and Tables

**Table:** 

Abbreviation	Full name
NHL	Non-Hodgkin lymphoma
MM	Multiple myeloma
TIL	Tumor-infiltrating lymphocyte
TCR	T cell receptor
DCs	Dendritic cells
HLA	Human leukocyte antigen
IP-MS	Immunoprecipitation-mass spectrometry
TAAs	Tumor associated antigens
ADCC	Antibody dependent cellular cytotoxicity
TSAs	Tumor-specific antigens
MHC	Major histocompatibility complex
pMHC	The peptide and MHC complex
NGS	Next generation sequencing
WES	Whole-exome sequencing
ATCC	American tissue cell culture
DMEM	Dulbecco's Modified Eagle's medium
DMSO	Dimethyl sulfoxide
FA	Formic acid
RLU	Relative light unit
FDR	False discovery rate
PBMCs	Peripheral blood mononuclear cells
ORF	Open reading frame
CDS	Coding sequence
DDA	Data-dependent acquisition
MAE	Mild acid elution
CAA	Confident amino acid
fLuc	Firefly luciferase
MFI	Mean fluorescence intensity
PMA	Phorbol myristate acetate
SA-PE	Streptavidin-PE
PSM	Peptide-spectrum matches
ppm	Parts per million
RT	Retention time
