# Supplementary material for: Rapid and direct discovery of functional tumor specific neoantigens by high resolution mass spectrometry and novel algorithm prediction
Source: Cell Insight. 2025 May 12;4(3):100251. doi: 10.1016/j.cellin.2025.100251 (PMC12179604; doi:10.1016/j.cellin.2025.100251)
Supplement: Multimedia component 1 [file mmc1.pptx]

## Slide 1
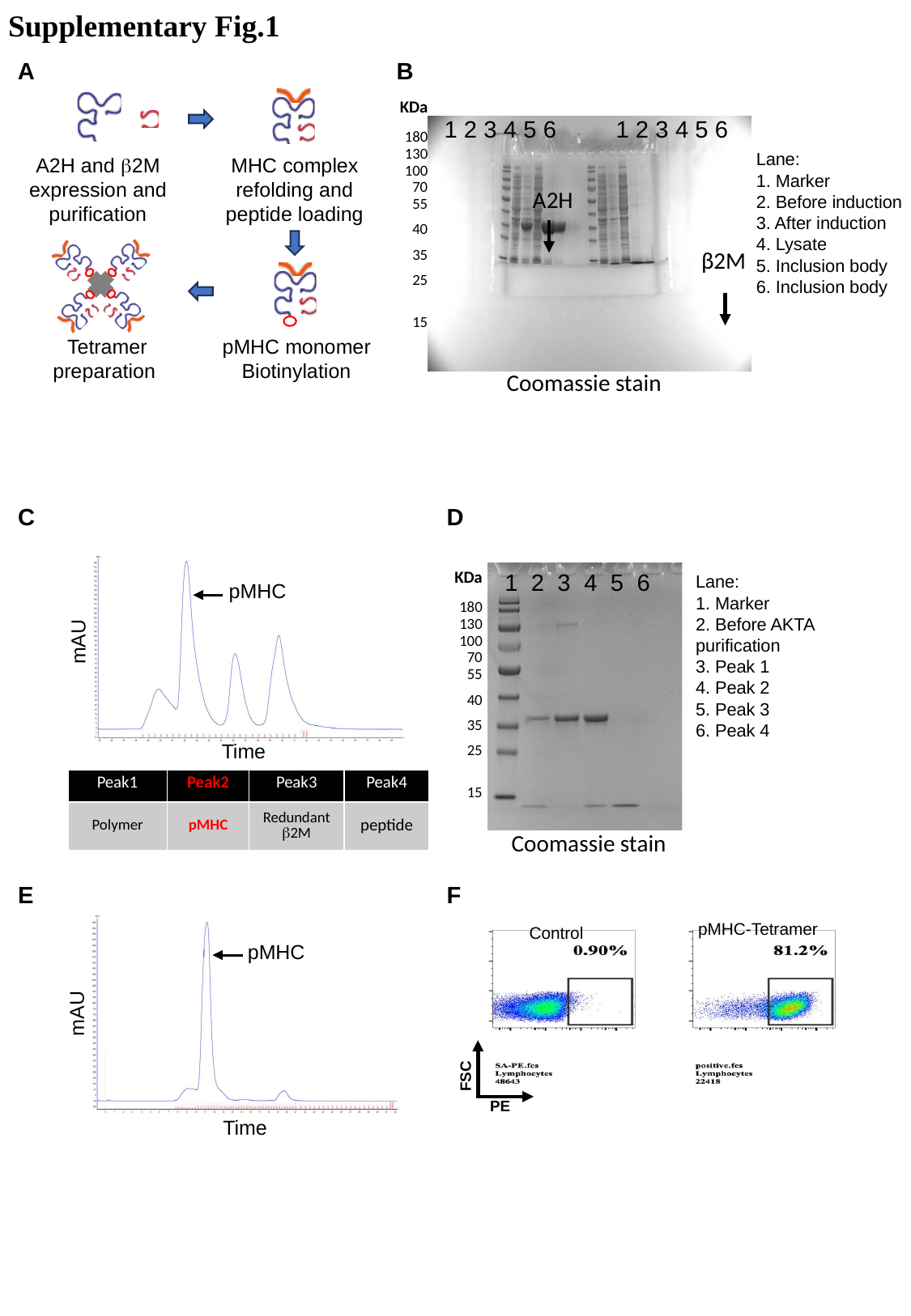

Supplementary Fig.1
A
B
KDa
180
130
100
70
55
40
35
25
15
1 2 3 4 5 6
1 2 3 4 5 6
A2H
Coomassie stain
Lane:
1. Marker
2. Before induction
3. After induction
4. Lysate
5. Inclusion body
6. Inclusion body
β2M
A2H and b2M expression and purification
MHC complex refolding and peptide loading
Tetramer preparation
pMHC monomer
Biotinylation
C
D
pMHC
mAU
Time
KDa
180
130
100
70
55
40
35
25
15
1 2 3 4 5 6
Lane:
1. Marker
2. Before AKTA purification
3. Peak 1
4. Peak 2
5. Peak 3
6. Peak 4
| Peak1 | Peak2 | Peak3 | Peak4 |
| --- | --- | --- | --- |
| Polymer | pMHC | Redundant b2M | peptide |
Coomassie stain
E
F
pMHC
mAU
Time
pMHC-Tetramer
Control
FSC
PE

## Slide 2
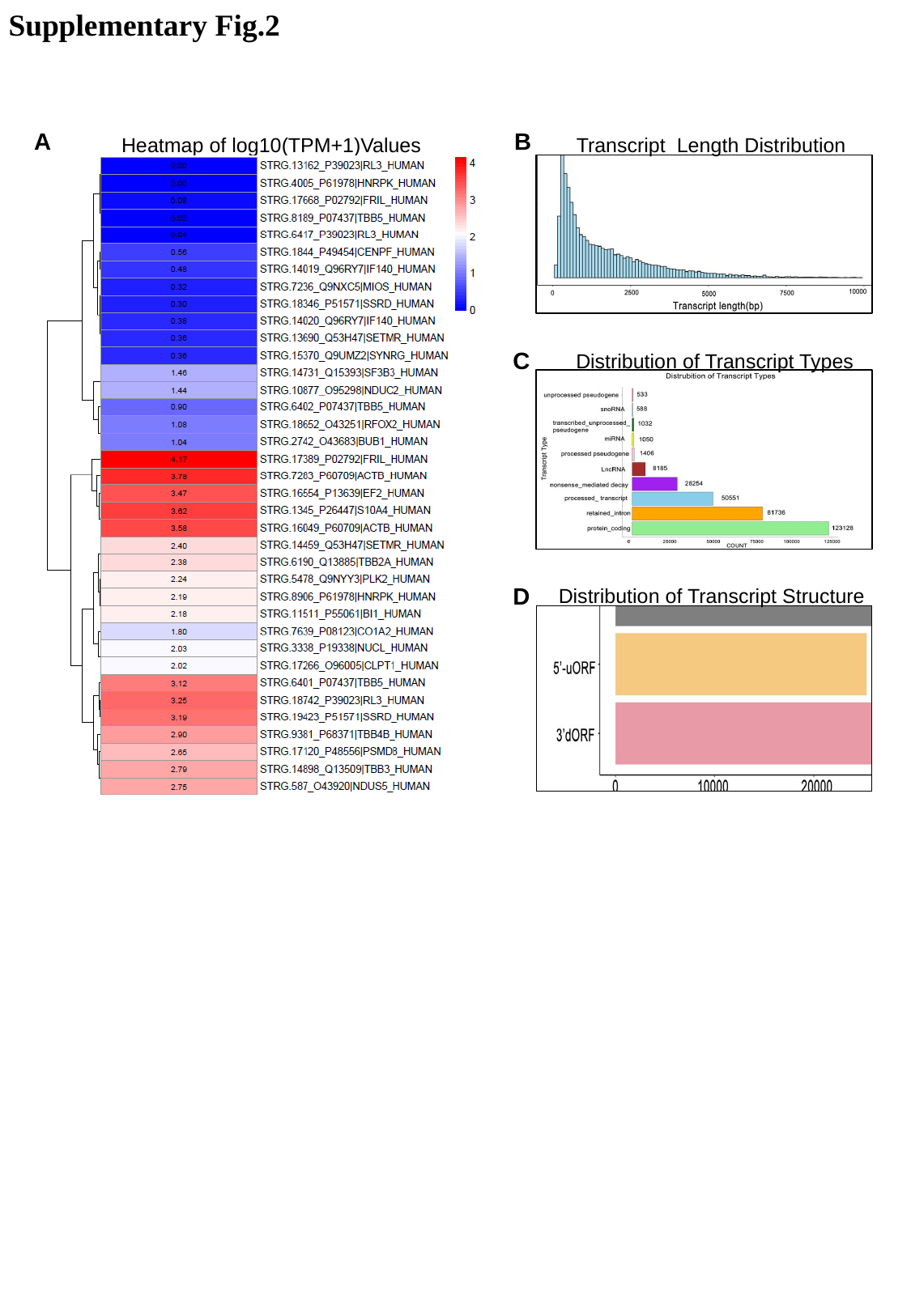

Supplementary Fig.2
A
B
Heatmap of log10(TPM+1)Values
Transcript Length Distribution
C
Distribution of Transcript Types
D
Distribution of Transcript Structure

## Slide 3
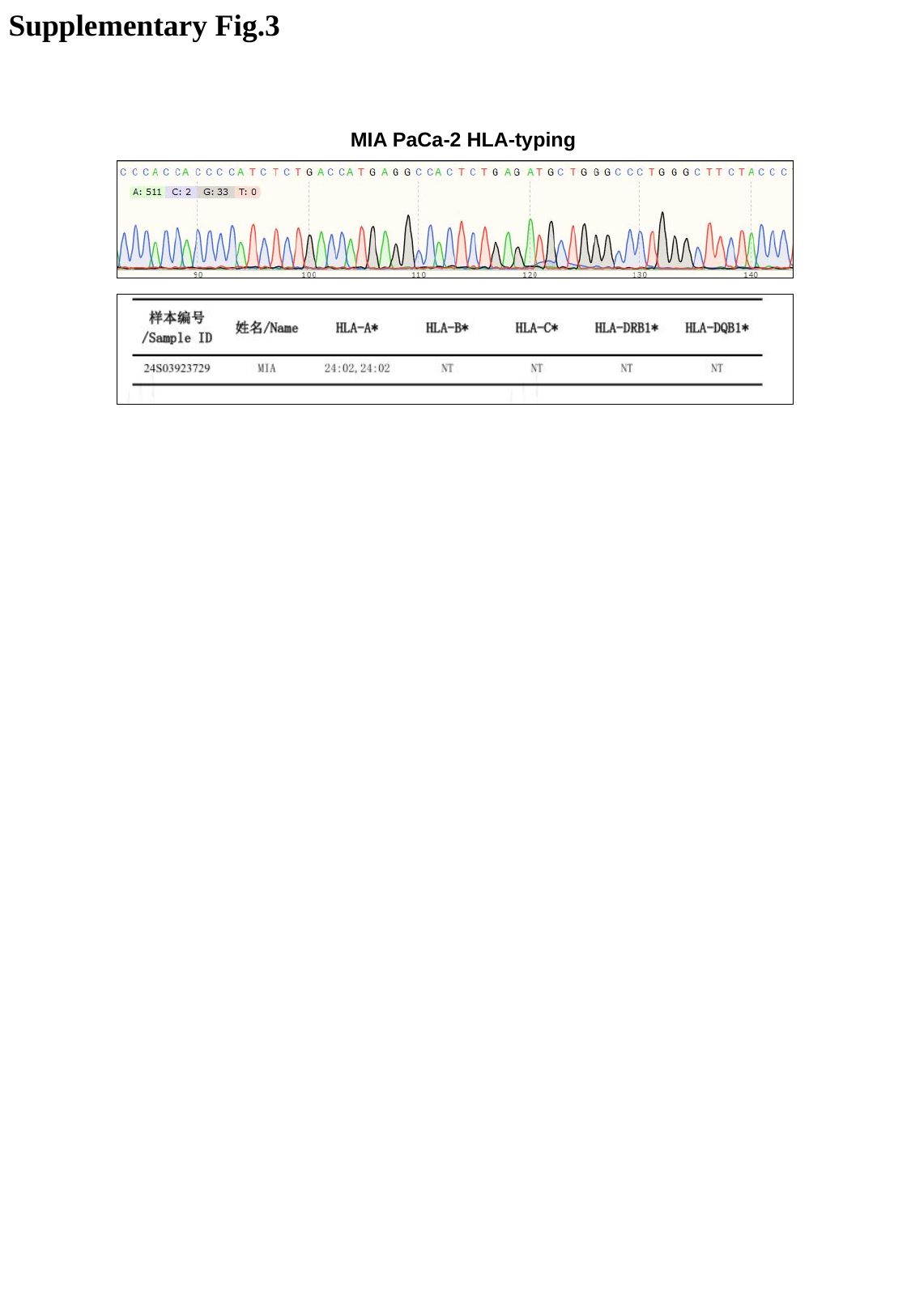

Supplementary Fig.3
MIA PaCa-2 HLA-typing

## Slide 4
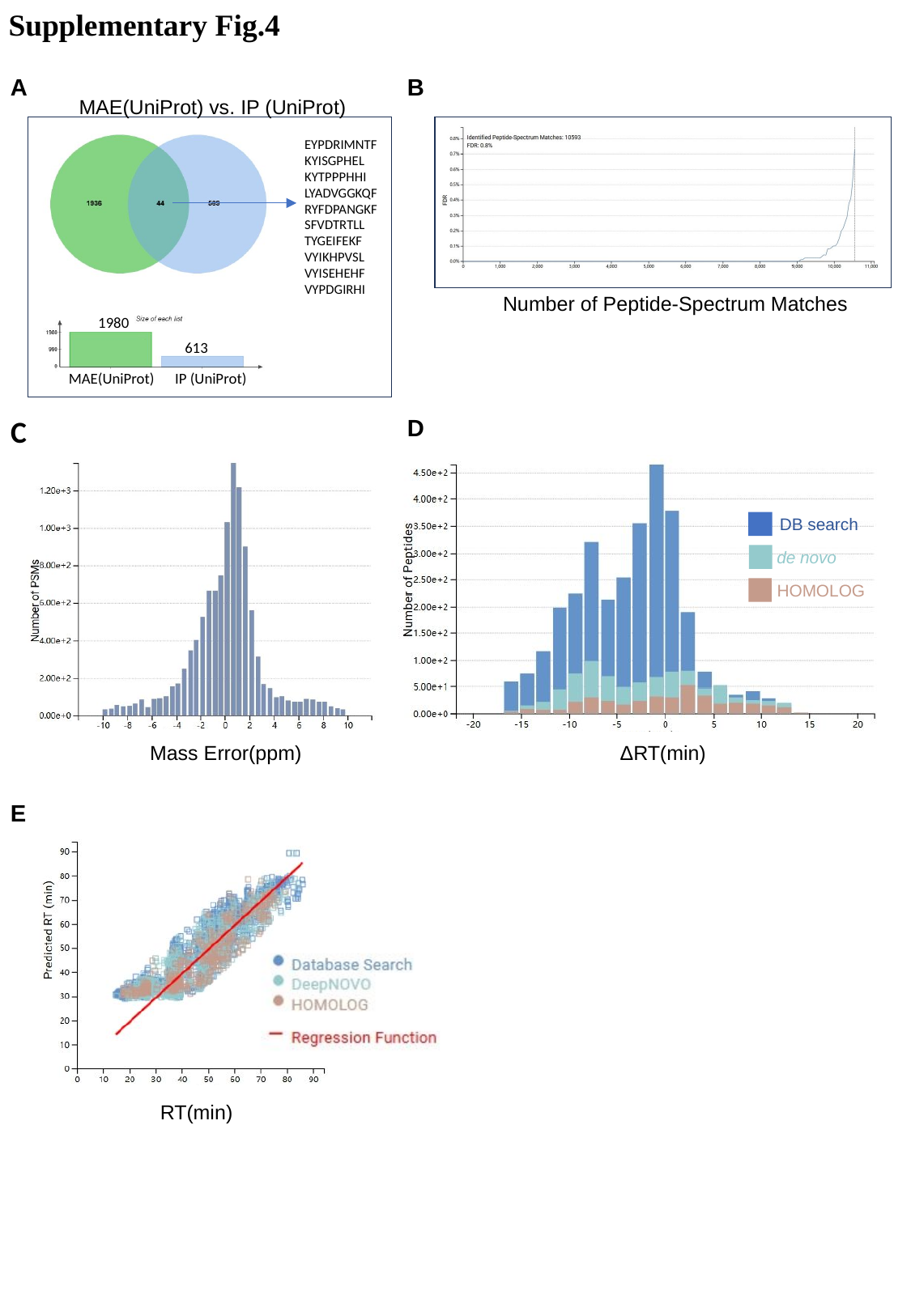

Supplementary Fig.4
A
B
MAE(UniProt) vs. IP (UniProt)
Number of Peptide-Spectrum Matches
EYPDRIMNTF
KYISGPHEL
KYTPPPHHI
LYADVGGKQF
RYFDPANGKF
SFVDTRTLL
TYGEIFEKF
VYIKHPVSL
VYISEHEHF
VYPDGIRHI
1980
613
MAE(UniProt) IP (UniProt)
C
D
DB search
de novo
HOMOLOG
Mass Error(ppm)
ΔRT(min)
E
RT(min)

## Slide 5
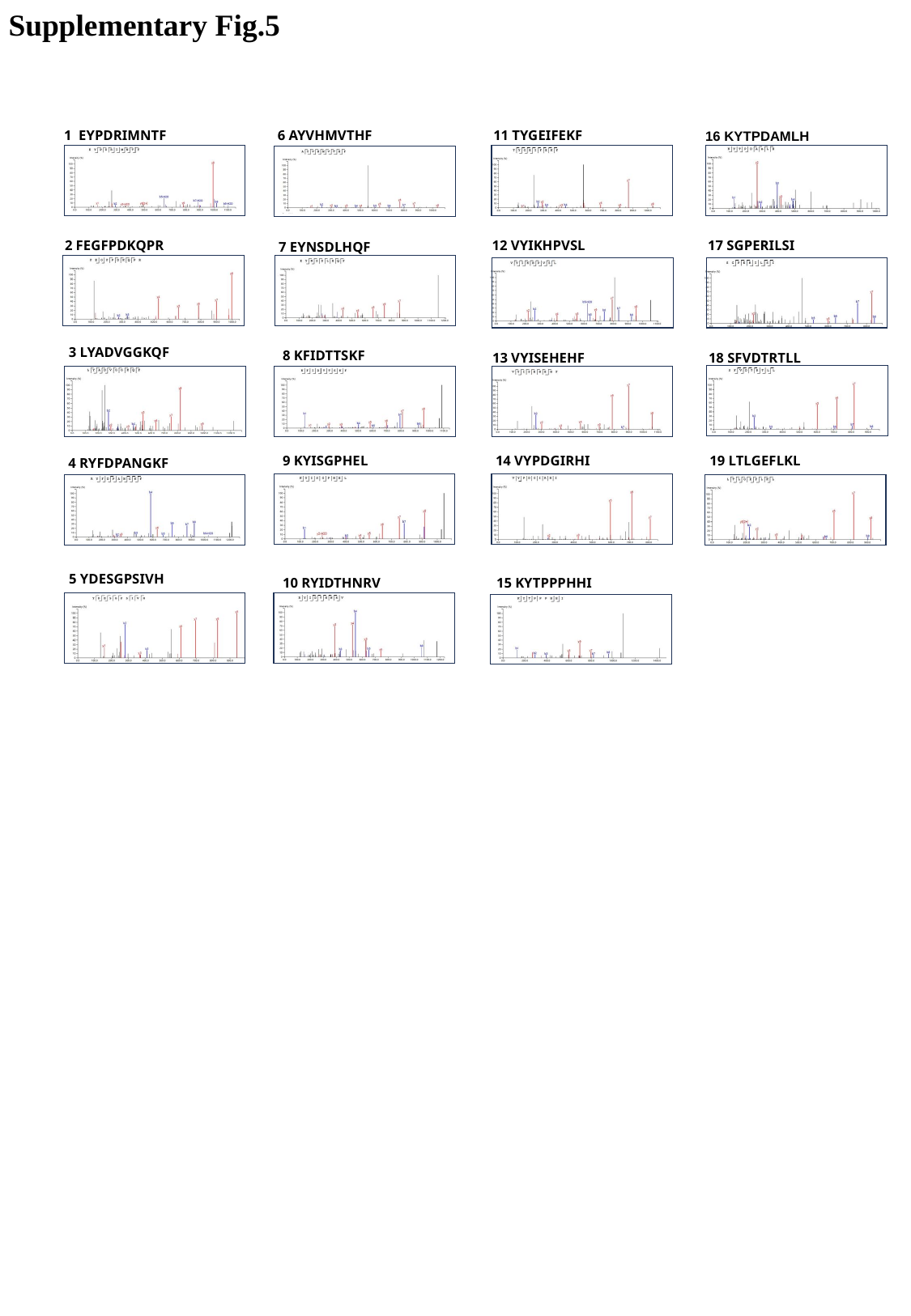

Supplementary Fig.5
1 EYPDRIMNTF
6 AYVHMVTHF
11 TYGEIFEKF
16 KYTPDAMLH
12 VYIKHPVSL
17 SGPERILSI
2 FEGFPDKQPR
7 EYNSDLHQF
3 LYADVGGKQF
8 KFIDTTSKF
13 VYISEHEHF
18 SFVDTRTLL
9 KYISGPHEL
14 VYPDGIRHI
19 LTLGEFLKL
4 RYFDPANGKF
5 YDESGPSIVH
10 RYIDTHNRV
15 KYTPPPHHI

## Slide 6
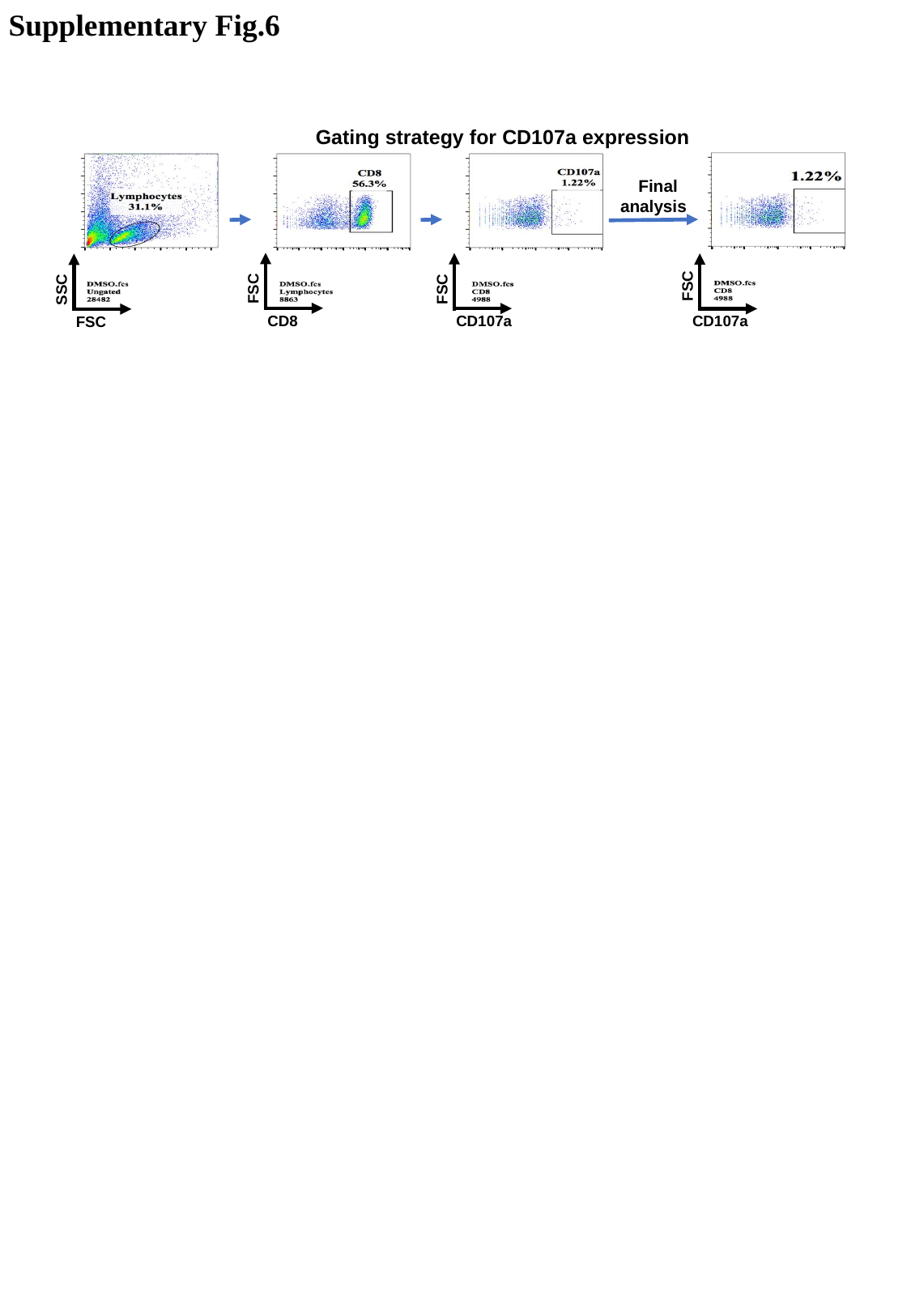

Supplementary Fig.6
Gating strategy for CD107a expression
Final analysis
FSC
FSC
FSC
SSC
CD8
CD107a
CD107a
FSC

## Slide 7
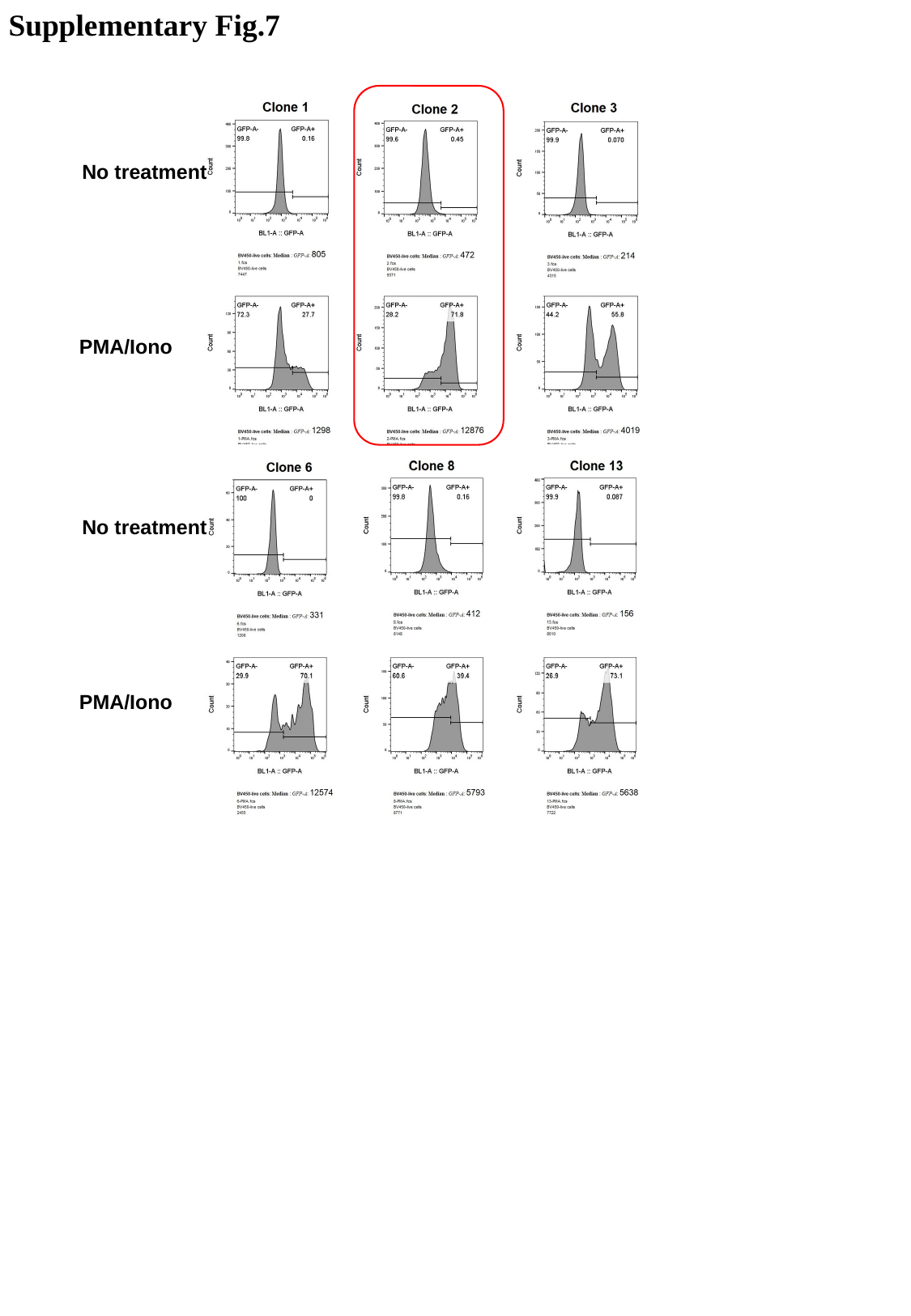

Supplementary Fig.7
No treatment
PMA/Iono
No treatment
PMA/Iono
